# Machine learning models for hydrogen bond donor and acceptor strengths using large and diverse training data generated by first-principles interaction free energies

**DOI:** 10.1186/s13321-019-0381-4

**Published:** 2019-09-11

**Authors:** Christoph A. Bauer, Gisbert Schneider, Andreas H. Göller

**Affiliations:** 1 Department of Chemistry and Applied Biosciences, Swiss Federal Institute of Technology (ETH), 8093 Zurich, Switzerland; 2 Bayer AG, Pharmaceuticals, R&D, 42096 Wuppertal, Germany

**Keywords:** Computational chemistry, Density functional theory, Hydrogen bond strength, Free energy prediction, Cheminformatics

## Abstract

We present machine learning (ML) models for hydrogen bond acceptor (HBA) and hydrogen bond donor (HBD) strengths. Quantum chemical (QC) free energies in solution for 1:1 hydrogen-bonded complex formation to the reference molecules 4-fluorophenol and acetone serve as our target values. Our acceptor and donor databases are the largest on record with 4426 and 1036 data points, respectively. After scanning over radial atomic descriptors and ML methods, our final trained HBA and HBD ML models achieve RMSEs of 3.8 kJ mol^−1^ (acceptors), and 2.3 kJ mol^−1^ (donors) on experimental test sets, respectively. This performance is comparable with previous models that are trained on *experimental* hydrogen bonding free energies, indicating that molecular QC data can serve as substitute for experiment. The potential ramifications thereof could lead to a full replacement of wetlab chemistry for HBA/HBD strength determination by QC. As a possible chemical application of our ML models, we highlight our predicted HBA and HBD strengths as possible descriptors in two case studies on trends in intramolecular hydrogen bonding.

**Electronic supplementary material:**

The online version of this article (10.1186/s13321-019-0381-4) contains supplementary material, which is available to authorized users.

## Introduction

The hydrogen bond [1] (HB) is a key non-covalent interaction in biochemistry and medicinal chemistry [2–12]. It has been demonstrated that a single HB interaction can decide the potency of drug-like molecules for a target when all other interactions stay constant [13]. HB strength can be approximated by the experimental reaction Gibbs free energy ($\Delta G$) in the case of 1:1 complex formation when all other intermolecular interactions are small. Scales for hydrogen bond acceptor (HBA) and donor (HBD) strengths can be derived by using a common monofunctional reference donor/acceptor molecule.

Significant experimental work was already carried out in the 1960s, when HBA strengths were measured against 4-fluorophenol by Taft and co-workers [14]. A HBD strength scale for solvents was established in 1976 by the same group [15]. Abraham and co-workers established experimental scales of HBA and HBD strengths against various reference molecules [16–20]. At around the same time, Raevsky et al. developed HB scales using both enthalpies and free energies [21–23]. Their HYBOND database [24] is one of the largest HB databases to date. The Fourier Transform Infrared Spectroscopy (FTIR) based p*K*_BHX_ database [25] comprised approximately 1200 entries of experimentally measured HBA strengths. There, the majority of the values were based on 1:1 complex formation. For HBD strengths, a similar measure, the p*K*_AHY_ value was established [26], but for far fewer molecules, mainly alcohols [27].

HBA/HBD strengths predicted by Quantitative Structure–Property Relation (QSPR) models have involved quantum-chemical (QC) descriptors, among them orbital energies and other output of QC calculations [28, 29], the electrostatic potential [30–33], COSMO polarization densities [34, 35], and optimized geometries of 1:1 H-bonded complexes [36]. A recent approach by the group of Varnek involves training a support vector machine learning (ML) model on ISIDA fragment descriptors, which take into account both donor and acceptor sites [37, 38]. HBA/HBD strengths were also computed by supramolecular QC. Gas phase models of 1:1 complexes yielded H-bonding energies that correlated well with experiment [39–42] as did computations on HBD strengths in implicit solvent [43]. Recently, we presented our own approach using ML with atomic radial descriptors [44–46] and QC computations [47].

Exploring the chemical space using QC methods has very recently come into focus [48]. By generating data points in silico and training ML models on them, larger areas of chemical space can be covered in smaller time scales. Examples include bond dissociation energies [49, 50], dipole moments [51], and partial charges [52, 53].

Our work ties in directly with this concept: We quantum chemically compute Gibbs free energies of HB formation in CCl_4_, with the aim of fully substituting experiment. Our reference HBD is 4-fluorophenol and our reference HBA is acetone. Both were used extensively in experimental studies. Figure S1, found in Additional file 1 the illustrates the reaction types used in our study.

## Methods

### Data sets

#### Experimental data sets for quantum chemistry validation

The pK_BHX_ database [25] contains experimental free energies for hydrogen bond acceptor molecules. It uses the 4-fluorophenol scale. The data set was obtained from the authors and 425 monofunctional compounds serve as experimental validation set. These compounds comprise oxygen, nitrogen, and unpolar (alkenes, halides, etc.) HBA moieties to cover as broad a chemical space as possible.

For the experimental donor strengths, we used a data set obtained from Varnek [38], which we call the Strasbourg database below and extracted all 58 data points containing acetone as the reference acceptor in the solvent CCl_4_ from the training set.

#### Generation of hydrogen bonding fragments for the quantum chemical databases

**Fig. 1:**
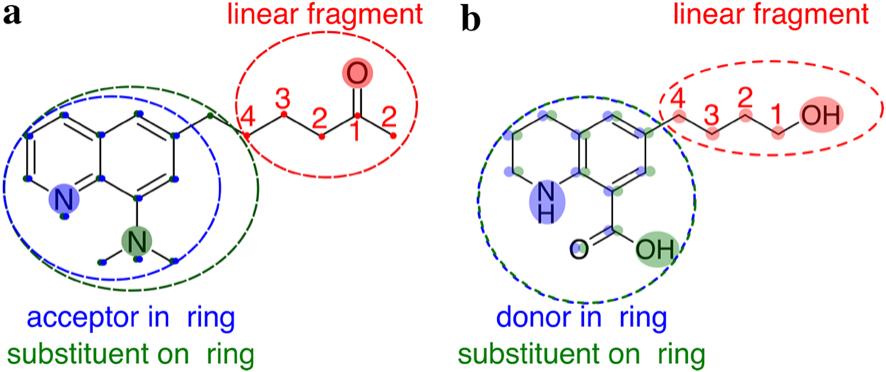
Depiction of the fragmentation strategy to obtain fragments containing **a** acceptor and **b** donor functionalities

Molecular fragments containing HBA/HBD moieties were generated using the following strategy, as depicted in Fig. 1.

1. Define donor and acceptor atoms:
  - Define HBA sites: Every oxygen, every nitrogen except if bound to oxygen.
  - Define HBD functions: R–OH (alcohols), R-NH_2_ (primary amines), R^1^–NH–R^2^ (secondary amines, heterocycles), R–SH (thiols), R–C≡C–H (alkynes).
2. Iterate over all HBA sites. Get the substructure up to the 4th shell of topologically connected atoms. Three cases are defined:
  - Chain fragment: Atoms around the HBA site are not in any ring up to the third shell. If fourth shell atoms are in a ring, the atom type is changed.
  - Ring + sidechain fragment: At least one atom within the third shell around the HBA site is part of a ring. The whole ring is taken in addition to the sidechain, which extends to the fourth shell.
  - Ring fragment: The HBA site is in a ring. The whole ring system and any side chains up to the fourth shell are taken.

This strategy, which is similar to a functional group identification scheme developed by Peter Ertl [54], was implemented in rdkit 2017.09.1 [55]. The unique fragment incidences were counted by comparison of canonical SMILES strings. Importantly, all molecules were kekulized (i.e., only single, double and triple bond types were used, no aromatic bond types), which ensured that heterocyclic compounds, for which aromaticity is sometimes ill-defined within cheminformatics frameworks, were treated correctly. The QM-derived partial charges are nevertheless based on aromatic bonds.

The resulting unique acceptor and donor fragments were subjected to a selection procedure: Only organic fragments (atoms H, C, N, O, F, Cl, S, Br, I) were accepted. Further criteria for selection were the number of rings (less than four), the corrected molecular weight being below 300 D [56], and the number of donors/acceptors in any fragment (less than four). The resulting subset of unique fragments was first grouped into six classes for acceptors as defined by atom type (O, N) combined with fragment type (chain, ring + sidechain, ring) and 9 out of 12 thinkable classes in case of donors as defined by atom type (O, N, S, C_sp_) combined with fragment type (not occurring were not unexpectedly O-ring, C-ring, S-ring). Each such class was subjected to a clustering procedure using the Pipeline Pilot [57] component “cluster molecules” with MDL public keys fingerprints, Tanimoto distance metrics, maximum dissimilarity and optimized for speed and memory. NumberOfClusters was set to 1/25 of the number of fragments of each class, and we kept at maximum 30 fragments including the three most central compounds from each.

#### Energy values

We used energy values in units of kJ mol^−1^ as our target values. The experimental hydrogen bonding free energies for complex formation in the p*K*_BHX_ data set were measured by a infrared (IR) spectroscopic method: The shift in absorption induced in the hydrogen-bonded complex was used to determine the equilibrium constants and thereby the free energies. CCl_4_ was used as the solvent partly because it was IR transparent [25]. The Strasbourg data [38] were collected from a variety of different primary sources. We pointed out in our previous paper that comparing entries for molecular duplicates between the two different sources had a root mean square error (RMSE) of approximately 2 kJ mol^−1^ [47].

#### Quantum chemistry

For each of the generated fragments, we calculated reaction free energies $(\Delta G)$ in solution. The computational protocol comprised the following steps:

1. Generation of one 3D conformer of each donor or acceptor molecule and the reference donor and acceptor molecules 4-fluorophenol and acetone by the ETKDG method [58] using rdkit, Version 2017.09.1 [55].
2. GFN-xTB [59] semi-empirical QC single point computation including the generation of Foster–Boys localized molecular orbitals [60] and their charge centers for the acceptor molecules.
3. Generate one conformer for each HBA/HBD site with the reference donor 4-fluorophenol or the reference acceptor acetone:
  - For acceptor molecules: Placement of the donated hydrogen of 4-fluorophenol at a distance of 2.00 Å from the localized lone pair (LP) charge center at an angle of 180°. As our modelling approach is a single-structure strategy, the energetically higher (i.e. less stable) LP was taken.
  - For donor molecules: Placement of the donated hydrogen at a distance of 2.00 Å from an LP of acetone (isoenergetic orbitals) at an angle of 180°.
4. Constrained geometry pre-optimization of each complex structure with distance and angle constraints of 2.00 Å and 180°, respectively, using the MMFF94s [61–66] implementation [67] of Landrum and co-workers in rdkit, Version 2017.09.1.
5. Density Functional Theory (DFT) geometry optimization for acetone, 4-fluorophenol, each acceptor molecule, each donor molecule, and each pre-optimized complex at the PBEh-3c level of theory [68].
6. Calculation of rigid rotor/harmonic oscillator thermal corrections [69] *G*_RRHO,PBEh-3c_ for all species using the Hessian calculated at the PBEh-3c level of theory.
7. Single-point calculation at the dispersion-corrected PW6B95-D3(BJ)/def2-QZVP [70–74] level of theory (*E*_high-level DFT_). The use of dispersion corrections and a large basis set is needed for an accurate description of non-covalent interactions [75].
8. Implicit solvent calculation for the solvation free energies δ*G*_solv_ at the SMD/BP86-def2-TZVP [74, 76–78] level using CCl_4_ as the solvent.

The final reaction free energies in solution were thus calculated:

$$\begin{aligned} \Delta G_{sol,QC} &= \Delta E_{high - level DFT} + \Delta G_{RRHO,PBEh - 3c} \\ &\quad+ \Delta \delta G_{{solv, SMD \left( {CCl_{4} } \right)}}, \end{aligned}$$

with

$$\begin{aligned} \Delta E &= E\left( {complex} \right) - E\left( {molecule} \right) \\ &\quad- E\left( {reference \,donor \,or\, acceptor} \right). \end{aligned}$$

All DFT computations were carried out using Turbomole 7.0.2 [79] and Gaussian 09, Revision D.01 [80]. (SMD calculations) at ETH Zürich and at Bayer high-performance computing clusters.

#### Machine learning

We apply our previously developed [44, 45] radial atomic reactivity descriptors for the HBA and HBD sites, encoding the electronic and steric environment of an atom, together with Gaussian Process Regression (GPR) [81]. It provides a native estimate of the variance by taking into account the distance of the query to the training data in descriptor space. We used the GPR implementation of scikit-learn 0.19.1 [82] with a combined kernel function:

1 $$K = C * M + W,$$

where *C* is a constant (parameter optimization scale from 10^−3^ to 10^3^), M is the Matérn kernel function (with fixed parameter ν; manually scanned at values of 1/2, 3/2 and 5/2, and length scale *l* optimization (from 10^−2^ to 10^2^),

2 $$M\left( {x_{i} ,x_{j} } \right) = \sigma^{2} \left( {1 + \gamma \sqrt 3 d\left( {\frac{{x_{i} }}{l},\frac{{x_{j} }}{l}} \right)} \right)exp\left( { - \gamma \sqrt 3 d\left( {\frac{{x_{i} }}{l},\frac{{x_{j} }}{l}} \right)} \right),$$

where *x*_*i*_*,x*_*j*_ are the data points, *d* is their distance, and γ is a fixed non-negative parameter.

*W* is a white kernel with an added noise level of 0.05. The kernel function involving the Matérn kernel turned out to yield the best-performing models. However, we also scanned over different kernel functions and compared to other ML regression methods.

## Results and discussion

### Generated acceptor and donor Fragments

276,004 molecules were extracted from the ChEMBL23 database [83] and washed. Only compounds with at least one activity value below one micromolar against at least one target were kept. Applying our fragmentation strategy yielded 162,732 unique HBA and 50,268 unique HBD fragments. The top 10 acceptor fragments with their incidences and a selection of diverse and frequent donor fragments are shown in Tables S1 and S2, found in Additional file 1, respectively. An aliphatic carboxylic acid moiety tops both lists. It was found 5882 times as an acceptor, and 2941 times as a donor due to the designation of both its oxygens as possible acceptor sites. This is due to our choice to only compute neutral fragments. The carbonyl oxygen of the carboxy group has an acceptor strength of around 10 kJ mol^−1^.

The subsequent filtering and selection steps afforded 3326 acceptor fragments containing one to four possible acceptor sites, and 1088 donor fragments containing one or two possible donor sites. The acceptors were therefore more abundant and chemically more diverse than the donors, which was to be expected under our conditions since there were a lot of heterocyclic compounds in the original data set, which contained significantly more acceptors than donors. With those molecules at hand, we moved to compute $\Delta G_{sol,QC}$ for each contained HBA/HBD site.

### Relation of quantum chemistry to experiment

**Fig. 2:**
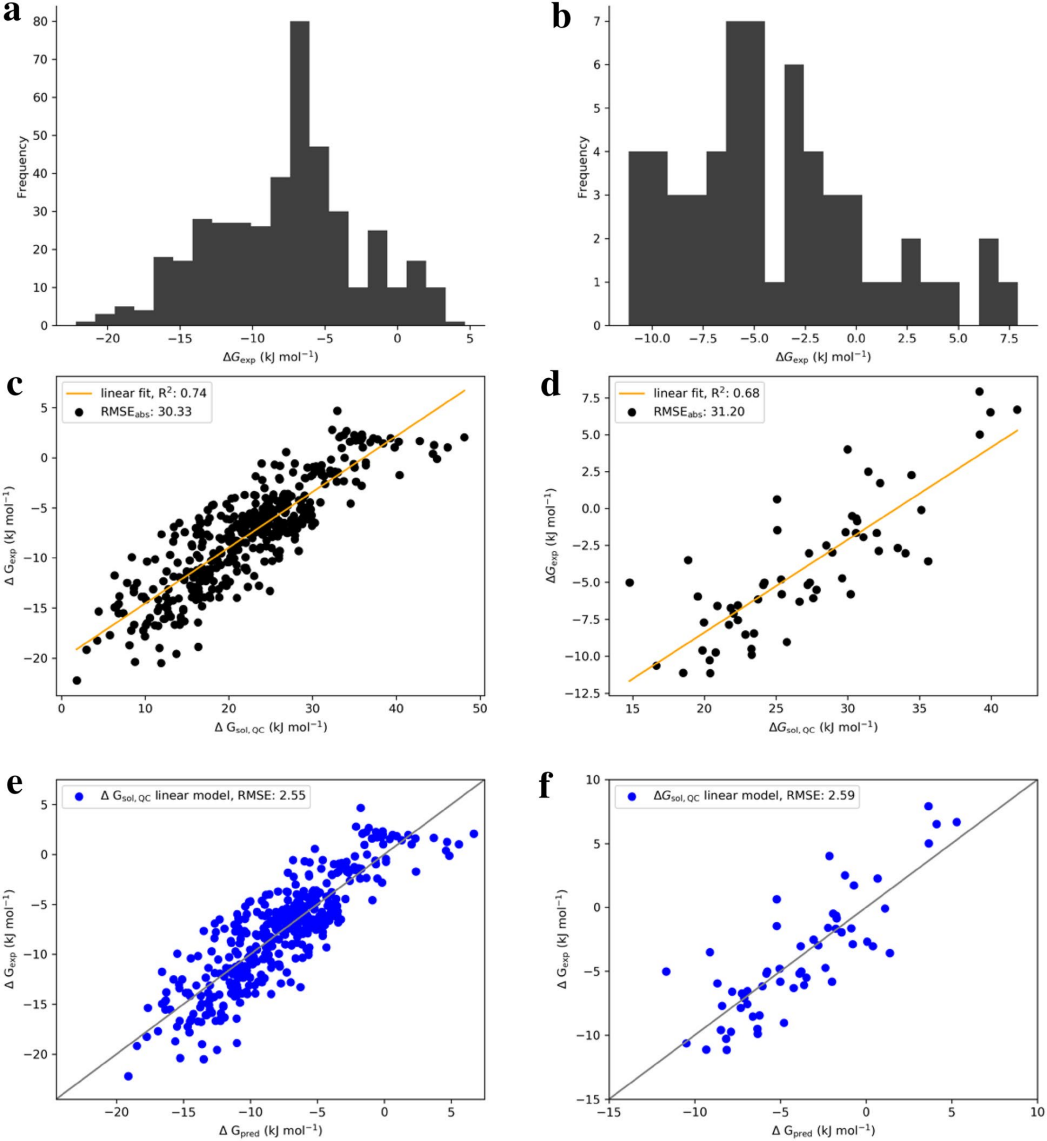
Experimental target value distributions for 425 acceptors (Gibbs free energies for 4-fluorophenol HB complex formation in CCl4, **a**) [25] and 58 donors [38] (Gibbs free energies for acetone HB complex formation in CCl_4_, **b**). Quantum chemical results for acceptors (**c**) and donors (**d**). Linear models for acceptors (**e**) and donors (**f**). The RMSEs are given in units of kJ mol^−1^

The first step was to calibrate the quantum chemical computations against experiment. A subset of 425 compounds from the p*K*_BHX_ set [25] was chosen as the experimental acceptor strength target value set. The experimental values of the acceptors ranged from − 20 to + 4 kJ mol^−1^. 58 compounds from the Strasbourg database were chosen for the donors [38]. The experimental values for donors ranged from − 10 to + 8 kJ mol^−1^. These distributions and calibration results were compiled for display in Fig. 2. For both donors and acceptors, the computed Gibbs free energies in solution were systematically off. This was corrected by fitting linear regression models for both the donor and acceptor compounds. The slopes and intercepts of the linear regression models were stable to internal cross-validation with less than 1% standard deviation in the slopes and below 5% standard deviation in the intercepts. For the acceptors, the target Gibbs free energy value of HB-formation with 4-fluorophenol was therefore defined as:

$$HBA \Delta G\,\left( {{\text{kJ mol}}^{ - 1} } \right) = \Delta G_{sol,QC} *0.56 - 20.12 {\text{kJ mol}}^{ - 1} .$$

For the donors, the target Gibbs free energy value of HB-formation with acetone was:

$$HBD \Delta G\,\left( {{\text{kJ mol}}^{ - 1} } \right) = \Delta G_{sol,QC} *0.63 - 20.94 {\text{kJ mol}}^{ - 1}.$$

Both linear regression models have very similar slopes of approximately 0.6 and intercepts of − 20 kJ mol^−1^. This apparently universal systematic quantum chemistry error for the computation of 1:1 HB complex formation in CCl_4_ can be traced back to overly repulsive $\Delta G_{RRHO,PBEh - 3c} {\text{ and }} \Delta \delta G_{{solv, SMD \left( {CCl_{4} } \right)}}$ contributions. The $\Delta G_{RRHO,PBEh - 3c}$ error may arise because of anharmonic contributions, which are not taken into account in the RRHO approximation. The solvation contributions are weakly repulsive, which may arise from the SMD parametrization itself. We assume that the combination of these two error sources leads to the large absolute RMSEs of approximately 30 kJ mol^−1^ of $\Delta G_{sol,QC}$ to experiment. Applying the linear models shown above, the RMSEs are reduced to 2.6 kJ mol^−1^ for both acceptors and donors. Thus, we call the $\Delta G \left ( {{\text{kJ mol}}^{ - 1} } \right)$ target values quantum-chemically derived instead of quantum chemical. A detailed analysis of the systematic error of HB formation prediction is beyond the scope of this work, which may, however, guide theoretical chemists in future method development.

### Quantum-chemically derived databases

6000 acceptor-4-fluorophenol complexes and 1650 donor-acetone complexes were quantum chemically computed. 4426 Gibbs free energies for HBA strengths and 1036 Gibbs free energies for HBD strengths were obtained. For each of these values, all the necessary quantum chemical calculations converged. The most prevalent reason for exclusion from the database was a failed PBEh-3c geometry optimization. For the acceptors, no X–H distance greater than 2.40 Å in the optimized complex structure was allowed and for the donors no X–H distance greater than 2.65 Å was allowed. Other reasons for exclusion of data points were (i) DFT convergence failures at any level and (ii) imaginary normal modes with a frequency more negative than − 50 cm^−1^, which is indicative to incomplete structure optimizations. This corresponded to a loss of 26 percent for the selected fragment HBA sites and 37 percent of the selected fragment HBD sites. Compared to the experimentally available data, the final numbers of entries in the acceptor database were four times as many (4426 vs. approximately 1200 in the full p*K*_BHX_ database). For the donors, that factor was even higher, as previously only a few dozens of points were available on a single scale.

Having started from clustered fragment structures according to their chemical diversities, such a loss rate is manageable because sufficient chemical diversity for application (vide infra) is retained. Nevertheless, future work will certainly include amendment and expansion of the databases.

**Fig. 3:**
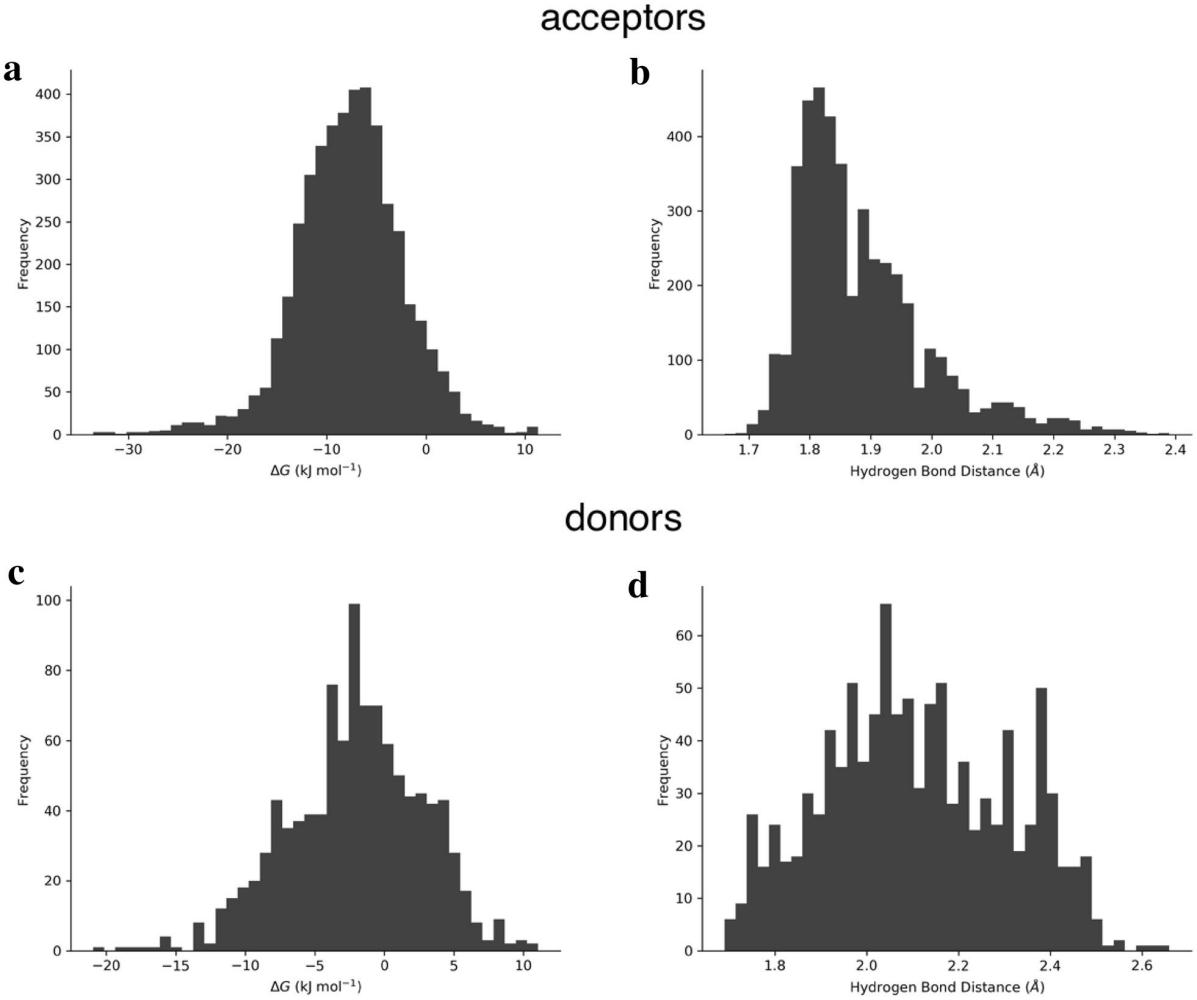
Distributions of quantum chemically derived free energies for the acceptor and donor databases (**a**, **c**) and distributions of hydrogen bond distances (X–H) at the PBEh-3c level of geometry optimization for the acceptor and donor databases (**b**, **d**)

The distributions of free energy values and X–H distances for the acceptor and donor databases are shown in Fig. 3. The acceptor database shows a nearly normal distribution of free energy values. This is expected because (i) we only took N and O as acceptors and (ii) we selected them to cover a variety of chemical space even within their functional group chemical spaces. Therefore, there are stronger and weaker carbonyls, amines, etc. among the acceptors, yielding a bell-shaped histogram for the free energies. The X–H distances reflect the different types of acceptors, because less polar HBA moieties like ethers have a systematically higher hydrogen bond distance, leading to enhanced population of distance values around 2.1 Å (Fig. 3b). For the donor database, the findings are similar: The free energy histogram (Fig. 3c) is bell-shaped. The hydrogen bond distance distribution is broader, reflecting the varying HBD strengths. Detailed statistics split by functional groups are given in Additional file 1 in the respective documentations of the acceptor and donor databases. The information therein reveals the following: The free energy of 1:1 HBA complex formation with 4-fluorophenol is more favored energetically with a total mean of − 7.72 kJ mol^−1^ compared to the free energy of HBD complex formation with acetone with a total mean of − 2.08 kJ mol^−1^. For the acceptors, the nitrogen-heterocyclic acceptors (imidazoles, pyrazoles, etc.) show the most negative free energies (mean values more negative than − 10 kJ mol^−1^) and thus the strongest HBA strengths. Carbonyls are medium acceptors. Alcohols and unpolar groups like ethers are weak HBAs. This is in line with the observations on the p*K*_BHX_ database, where the same ranking is described by the authors [25]. Alcohols are, (unsurprisingly) the strongest donors, followed by pyrroles. Amides are medium strength donors and thiols are very weak HBDs with a positive mean free energy for HB formation. The result that OH groups are generally stronger donors than NH groups is also found in Abraham’s 1989 paper [16].

**Fig. 4:**
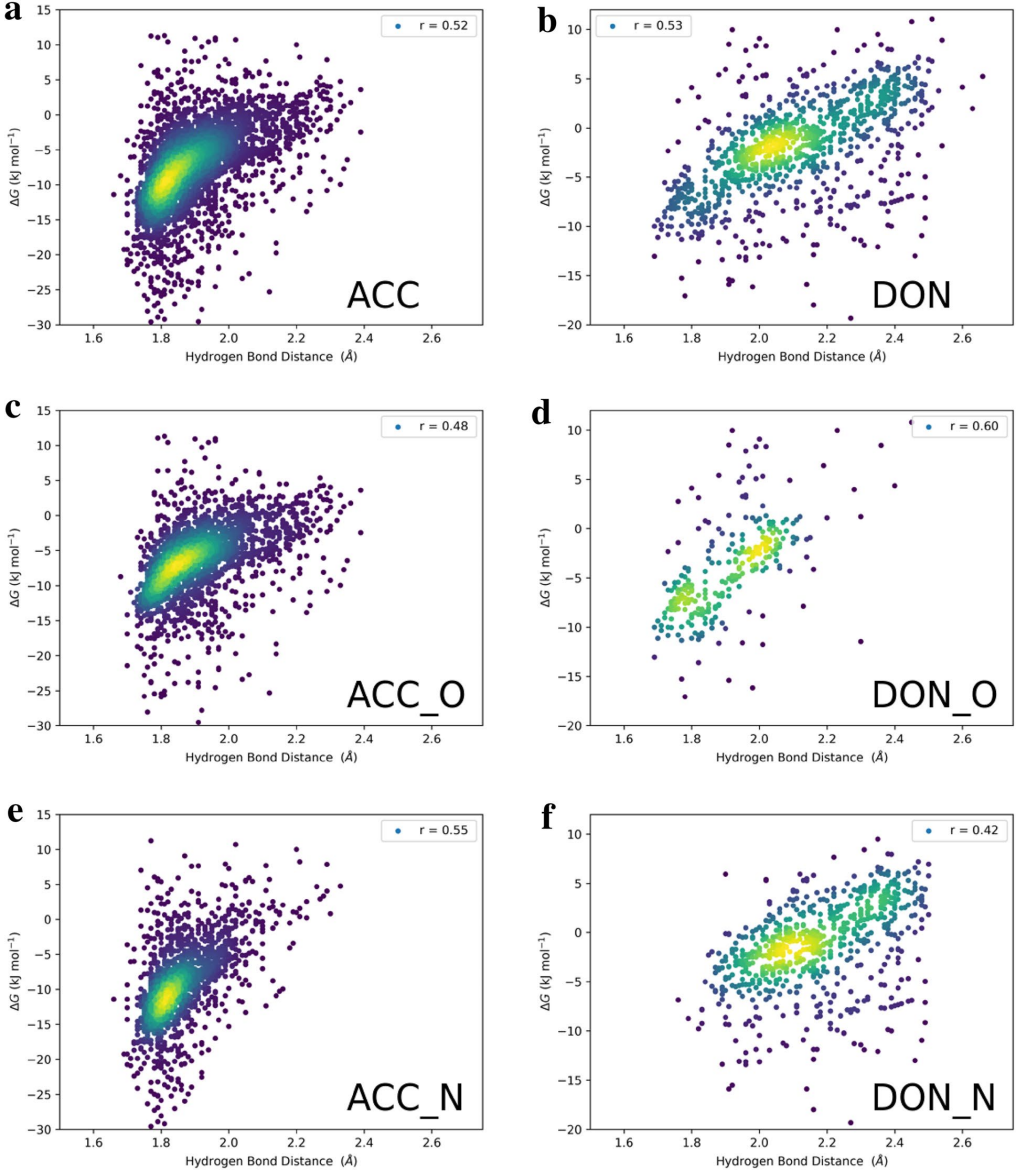
Hydrogen bond distances (HBA–H)) in units of Å for the total acceptor (**a**) and donor (**b**) databases vs the quantum chemically derived target values. **c**, **e** Show the same plots for only the oxygen and nitrogen acceptors, whereas **d**, **f** show the same plots for only the oxygen and nitrogen donors. The Pearson correlation coefficient (r) is given. The coloring of the points is according to point density: The lighter the color, the higher the point density

The HB distances in the PBEh-3c optimized complex structures are important indicators of the HBA/HBD strengths [36]. Figure 4 shows the HB distances against the QC-derived target values for the respective databases. For the acceptors (Fig. 4a), a funnel-like structure can be seen: The weaker the HBA strength (the more positive the free energy), the broader the distribution of distance values. This can be rationalized by the following example: A weak carbonyl acceptor will have a shorter hydrogen bond than a relatively strong ether acceptor, see also Fig. 4c, where only oxygen acceptors are plotted. However, the stronger the HBA gets, the less variety of HB distance there is, with the strongest HB formed at hydrogen bond (HBA–H) distances of 1.7 Å, see also Fig. 4e, where only nitrogen acceptors are plotted. There is also substantial correlation for the total data between the HB distances and the free energies (Pearson correlation *r* = 0.52). For the HBDs (Fig. 4b, d, f) the picture is similar: There is substantial correlation between the donor–acceptor distances and the free energies, in this case especially for the oxygen donors (alcohols and carbonic acids, Fig. 4d, *r* = 0.60) but it does not explain everything as seen by the worse correlation for the nitrogen donors (Fig. 4f, *r* = 0.42), indicating their larger chemical variation from amides to heterocycles to amines. This analysis is an important sanity check for the internal consistency of our databases.

**Fig. 5:**
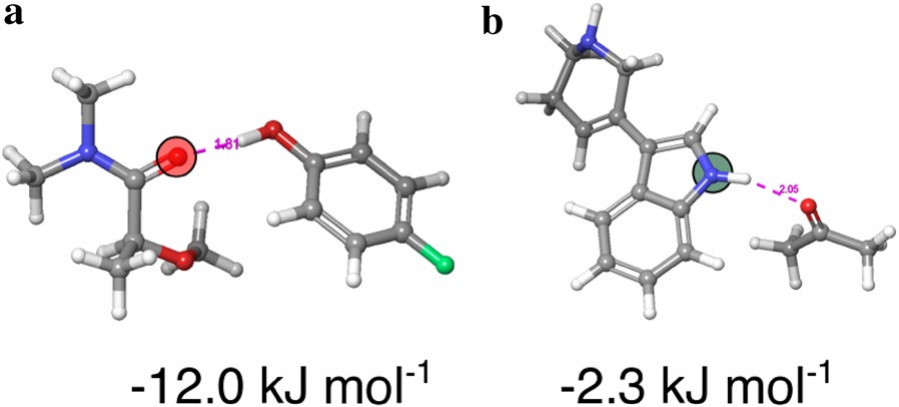
Representative 3D structures of the acceptor (**a**) and donor (**b**) complexes with the reference donor 4-fluorophenol (**a**) and acetone (**b**). The acceptor and donor atoms are marked with circles, and the associated QC-derived Gibbs free energies for complex formation are displayed

Two example entries of the QC-derived HBA/HBD strength databases are shown in Fig. 5. The carbonyl of the acceptor fragment has an associated HBA strength of − 12.0 kJ mol^−1^ and the pyrrole-like moiety of the donor fragment has an associated HBD strength of − 2.3 kJ mol^−1^. These examples illustrate the power of QC calculations because there are multiple sites in each fragment, which cannot necessarily be distinguished experimentally. Since free energies are in principle non-additive, it is also not trivial to assign a partial free energy value to each site. With QC, this can be done. The energetically most favored sites will be populated according to a Boltzmann distribution. Furthermore, to our knowledge, there are simply no experimental HBD strength values for 300 different amides as is the case in our HBD database.

### Machine learning model optimization and descriptor scan

In order to gauge the usefulness of our quantum-chemically derived databases, machine learning models were trained using our radial atomic reactivity descriptors. The trained models were evaluated in internal cross-validation (CV) and on test sets with experimental (not quantum chemical!) free energies. For the HBAs, the experimental test set consisted of 917 data points from the p*K*_BHX_ database [25] (converted to units of kJ mol^−1^). For the HBDs, we took the calibration set of 58 experimental values obtained from the Strasbourg database [38].

We performed two loops of scans: The first loop was for the optimal atomic descriptors for the HBA atoms and HBD atoms. The second loop was for the optimal machine learning models. A summary of descriptor types and kernels used in Gaussian Process regression, the best performing ML method, is found in Table 1. Complete tables on the performance of various descriptor types and other ML regression methods are found in Additional file 1. All descriptor elements involving partial charges were based on the GFN-xTB [59] computed CM5 [84] charges for the single conformer created by the method of Riniker and Landrum [58]. All atoms were used for descriptor creation, including the hydrogens.

**Table 1:** Radial atomic reactivity descriptors [45] for the HBA/HBD atoms used for machine learning and kernel functions in Gaussian Process Regression (GPR) as implemented in scikit-learn 0.19.1 [82]

Descriptor abbreviation	Description (for details, see our previous publication [45])
Sorted-shell	Charge shell descriptor with values sorted by Cahn-Ingold-Prelog rules
CS	Charge shell descriptor with average charge per shell
CRDF	Spatial charge radial distribution function
CACF	Spatial charge autocorrelation function (split into positive and negative parts)
MS	Mass shell; the elements are the sums of the masses of each shell
GACF	Topological charge autocorrelation function

GPR kernel function	Description
$C*RBF + W$	*RBF* = radial basis function (Gaussian)
$C*M + W$	*M* = Matérn kernel function (*v* scanned manually for values of 0.5, 1.5 and 2.5)
$C*RQ + W$	*RQ* = rational quadratic function

The hyperparameters of the constant kernel (*C*) and the *RBF*, *M*, and *RQ* functions were optimized in their default ranges (10^−2^ to 10^2^ for length scales, 10^−3^ to 10^3^ for *C*), and the white kernel (*W*) was used with a noise value of 0.05

**Fig. 6:**
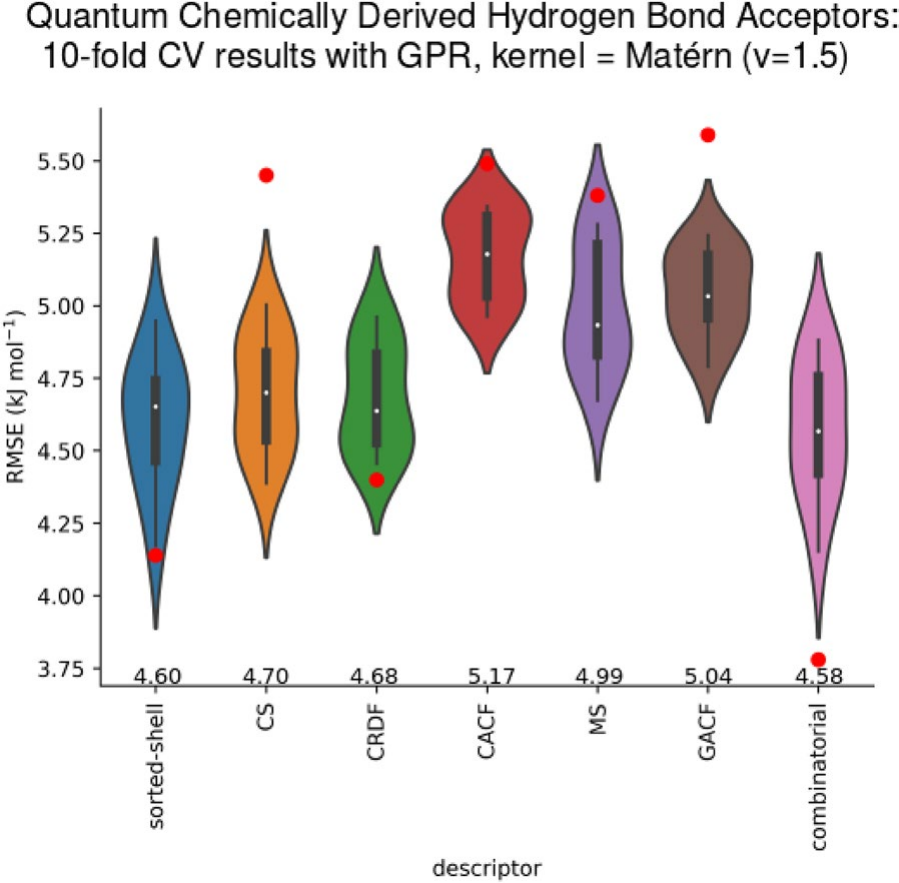
Violin plots: tenfold internal cross validation results and test set performances (red dots) for various atomic reactivity descriptor types with their respective best sets of descriptor parameters trained on 4424 QC-HBA data points. *RMSE*, root mean square error. The descriptor abbreviations are as follows: *CS* charge shell; *CRDF* = charge radial distribution function, *CACF* = spatial charge autocorrelation function, *MS* mass shell; *GACF* topological charge autocorrelation function, *combinatorial* combination of CACF, CS, and shorted-shell. The mean RMSEs of the tenfold CV results are indicated above the descriptor abbreviations. The red dots mark the RMSE on the experimental test set. The partial charge type used was CM5 for all atoms in all cases

For the acceptor database, the results of tenfold internal cross validation for various descriptor types are displayed in Fig. 6. GPR using the Matérn kernel (*v *= 1.5) was the prevailing ML method. Most descriptors failed at capturing the data adequately. The sorted shell descriptor performed best, followed by the radial distribution function descriptor. Both descriptors also had a better performance on the test set than in internal CV, as indicated by the red dots in Fig. 6.

The charge shell, spatial and topological charge autocorrelation and mass shell descriptors all perform badly, with an even worse performance on the test set. The combination of the shorted shell descriptor with the charge shell descriptor and the spatial charge autocorrelation function was chosen as the final combinatorial descriptor, for the exact parameter combination, see Additional file 1. Although the CS and CACF descriptors perform badly in internal cross-validation, the model obtained with the full training performed by far the best on the test set. The final combinatorial descriptor had 151 dimensions, which is appropriate for training on 4424 data points.

The bad performance of the descriptors in internal CV is mostly due to the large variety of chemical HBA space. More data points are required, and we hope that in the near future, either us or other members of the scientific community will be able to expand the databases that are available in full as Additional file 1 and compare their results to ours, which we view as an adequate beginning. The performance on the test set is discussed below.

**Fig. 7:**
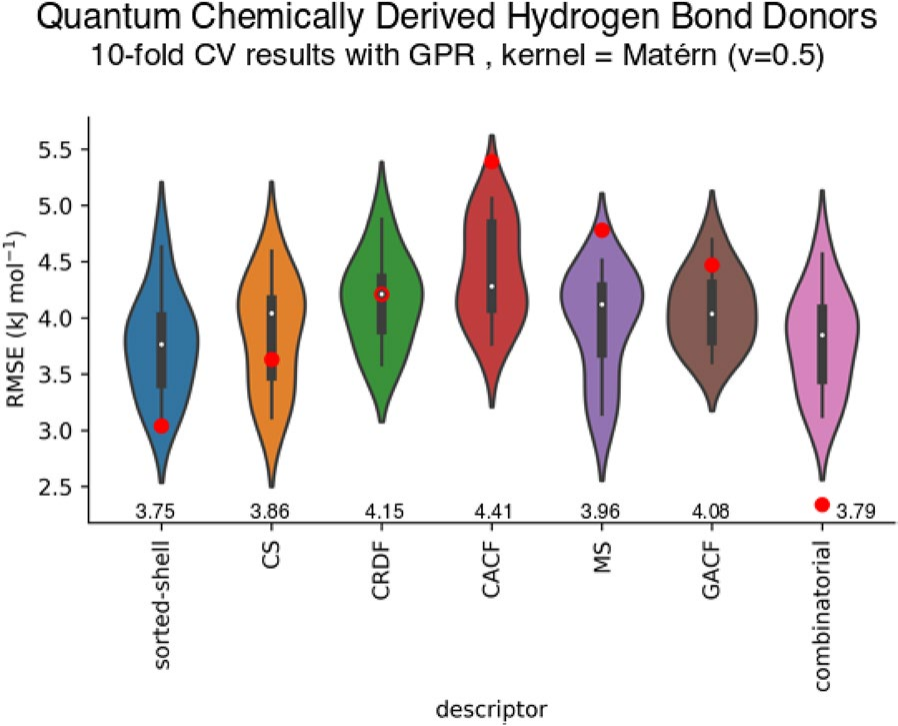
Violin plots: tenfold internal cross validation results and test set performances (red dots) for various atomic reactivity descriptor types with their respective best sets of descriptor parameters trained on 981 QC-HBD data points. *RMSE* root mean square error. The descriptor abbreviations are as follows: *CS* charge shell, *CRDF* charge radial distribution function, *CACF* spatial charge autocorrelation function, *MS* mass shell, *GACF* topological charge autocorrelation function, *combinatorial* combination of CACF, CS, and shorted-shell. The mean RMSEs of the tenfold CV results are indicated above the descriptor abbreviations. The red dots mark the RMSE on the experimental test set. The partial charge type used was CM5 for all atoms in all cases

For the donor database (results for the descriptor types with their respective best descriptor creation parameters shown in Fig. 7), 981 of the 1036 data points were used for training (for the others, there was some problem to compute all descriptors, e.g., the charge shell descriptor cannot be applied when there is no nth shell—the averaging leads to a division by 0) the best-performing ML models were the GPR models with a combined Matérn Kernel (*v *= 0.5). Among the descriptor types, the sorted-shell descriptor performed best both in tenfold internal cross-validation and on the test set. The charge shell descriptor, which averaged over the electronic environment of the HBA atoms, performed the second best. All other descriptor types performed worse, especially on the test set, marked by the red dots in Fig. 7. The charge radial distribution descriptor model had the same performance on the test set as in internal CV. The spatial charge autocorrelation function descriptor performed terribly on the experimental test set (the test set performance was in the upper range of its violin plot). Similar findings were true for the mass shell descriptor and the topological charge autocorrelation descriptor. The final descriptor is a combination of the spatial charge-autocorrelation function descriptor together with the sorted-shell and the charge-shell descriptors, for the exact combination of parameters, see Additional file 1. Although the charge-autocorrelation function descriptor performed badly on its own, in combination with the two other descriptor types, it led to the best performance on the test set of experimental free energies for HB formation with acetone. The final combinatorial descriptor had 115 dimensions, which is considered fair against the 981 training data points.

### Learning curves and applicability domain analysis

We analyzed also the learning curves and a perspective on the applicability domain via the GPR variance estimates for the HBA/HBD ML models using the final combinatorial descriptors.

**Fig. 8:**
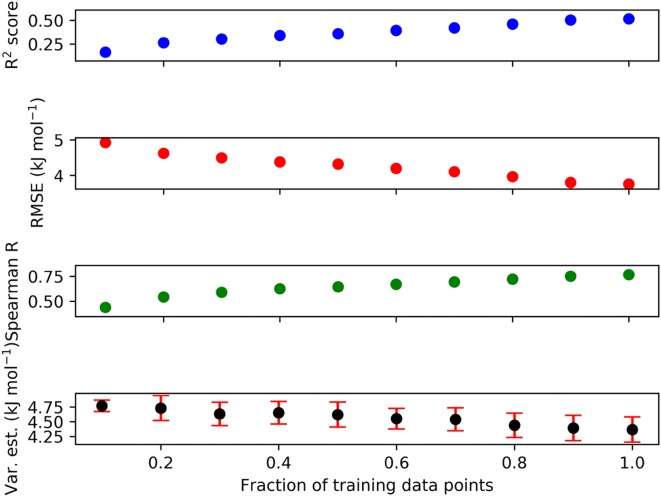
Learning curves including GPR variance estimates for the GPR (Matérn, v = 1.5, final 151-dimensional combinatorial descriptor) quantum chemically derived HBA database. Var.est = GPR variance estimate

Figure 8 shows the training performance of the GPR model for the HBAs using the final 151-dimensional descriptor against the fraction of QC target values. The learning curves were produced by increasing the fraction of training data points in 10% intervals from 0.1 to 1.0. The R^2^ score never reaches more than 0.50, which is an indication that further descriptor development or extension of the data is needed in the future as not even our best-performing descriptor can fully capture the atom space of acceptor atom environments available in molecules. The RMSE reaches its minimum value for the full training at around 3.7 kJ mol^−1^ (which is also the performance on the test set), which does not necessarily represent the optimally achievable accuracy. The Spearman correlation coefficient, which is a measure for the correct rank order of the data points, climbs continuously to a value of approximately 0.75. The GPR variance estimate (the 95% confidence interval inherently predicted by any GPR method) stays roughly constant around 4.5 kJ mol^−1^ until 70% of the training data points are included, and then continuously falls to a value of roughly 4.2 kJ mol^−1^. This analysis hints at the possibility of assessing the applicability domain of our HBA strength ML model: If the GPR variance estimate is significantly larger than 4.2 kJ mol^−1^, then the test data point may not be trustworthy.

**Fig. 9:**
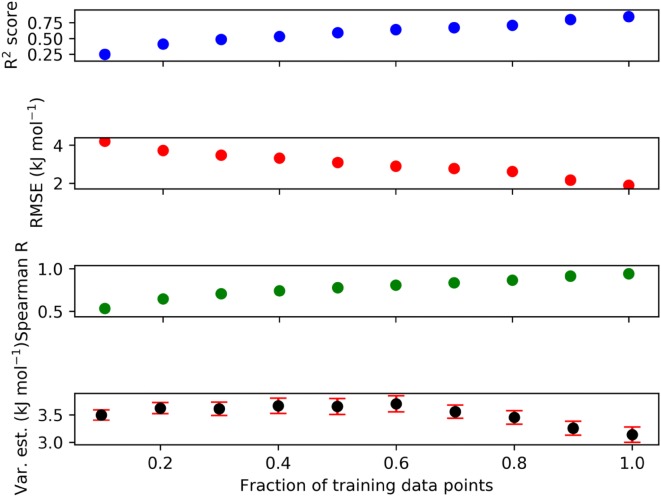
Learning curves including GPR variance estimates for the GPR (Matérn, v = 0.5, final 115-dimensional combinatorial descriptor) quantum chemically derived HBD database. Var.est, GPR variance estimate

Figure 9 provides the analogous analysis for the donors using the GPR model for the donors and the final combinatorial 115-dimensional HBD atom descriptor. For the hydrogen bond donors, performances are better across the board, which indicates that donor atom environments are less diverse than acceptor atom environments. The R^2^ score for the full training set reaches 0.75, and the RMSE is close to 2.0 kJ mol^−1^ The Spearman correlation coefficient climbs continuously to a value of almost 1, indicating almost perfect rank ordering for the fully trained GPR model on the training set. The GPR variance estimate for the HBDs stays roughly constant around 3.5 kJ mol^−1^ until 60% of the training data points are included, and then continuously falls to a value of roughly 3.2 kJ mol^−1^, providing an indication whether a predicted HBD strength is trustworthy or not. The significantly better performance for our ML models for the HBDs also shows in the evaluation on the test sets.

### Performance of machine learning models on experimental test sets

**Fig. 10:**
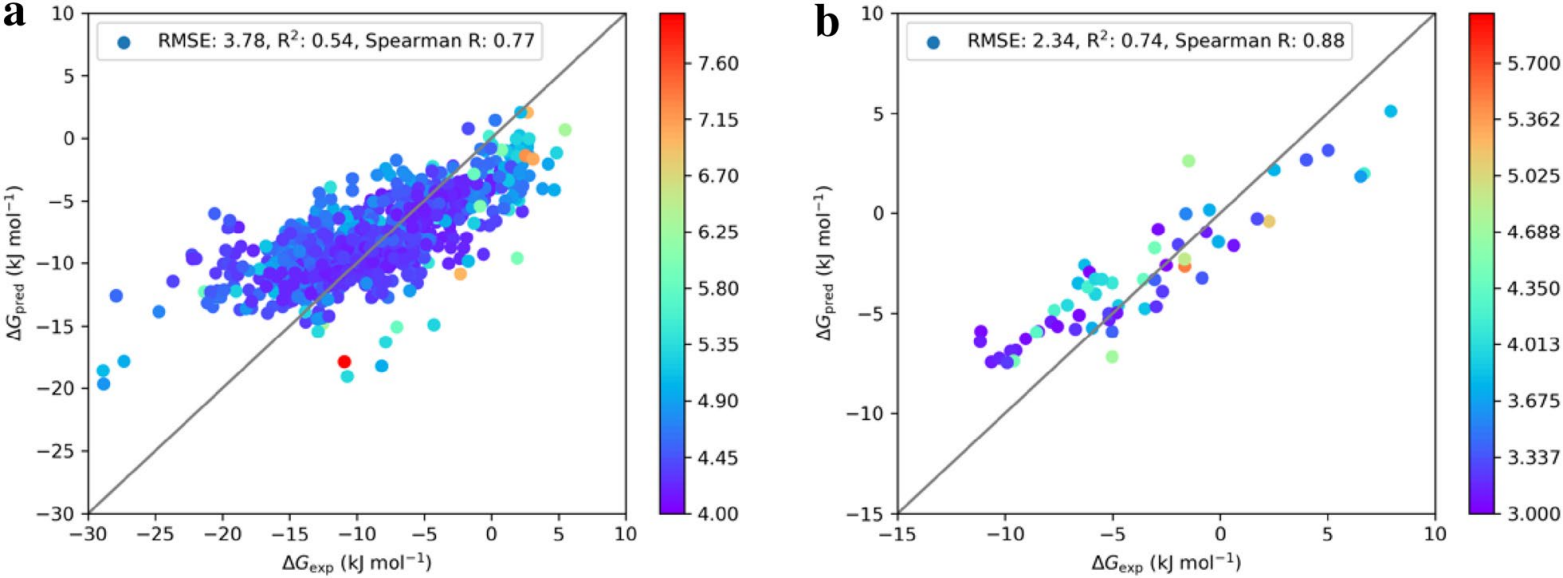
Performances of the HBA final 151-dimensional combinatorial descriptor GPR model trained on the quantum chemically derived free energies on the HBA test set (**a**) and of the HBD final 115-dimensional combinatorial descriptor GPR model on the HBD test set (**b**). The color bars show the GPR variance estimates for the respective models

Finally, we show the performances of both the acceptor and donor models using their respective final combinatorial descriptor/GPR combination. The test sets are 917 free energies of HB formation with 4-fluorophenol taken from the p*K*_BHX_ data base and the 58 free energies for HB formation with acetone that are used for calibration of the QC computations (in the absence of other experimental data). Figure 10 shows the performances of the final HBA and HBD models. The acceptor model predicts the HBA strength with an RMSE of 3.78 kJ mol^−1^, an R^2^ of 0.54 and a Spearman R of 0.77. The variance estimates range from 4.0 to 7.6 kJ mol^−1^ (although this high value is only reached for one data point in the test set, which is chemically apparently very different from the training data points). The target value distributions are found in Additional file 1: Figure S2.

This performance is considerably better than in internal cross-validation and comparable with the performance on the training set. We expect this to be due (i) error cancellation of experimental uncertainty and QC calculational error, and (ii) the p*K*_BHX_ contained acceptors have a lower chemical variety than the ones from the QM test set, even among only nitrogen and oxygen acceptors. In this light, predicting the experimental HBA strength at an expected accuracy of less than 1 kcal mol^−1^ using values that are created from thin air and first principles is at least a strong start. For the donors, the picture looks strikingly better. The final HBD model trained on QC-derived free energies predicts the experimental HBD strength with an RMSE of 2.34 kJ mol^−1^, an R^2^ of 0.74 and a Spearman R of 0.88. The variance estimates (3.0 to 5.0 kJ mol^−1^) are comparable to the one reached on the training set. Thus, our HBD strength model derived from QC computations is a fast and reliable means to assess HBD strengths.

With respect to the previously published models based on ISIDA fragment descriptors that can predict the strength of a hydrogen bond with in principle arbitrary HBA/HBD pairs [37, 38], our models have the following advantages: First, the data on which they are trained are easily extendable because they are computed using a robust quantum chemical protocol. Second, the GPR methodology gives an inbuilt estimate of the applicability of the models. Concerning the performance comparison on the test sets, we note that our final trained HBA model performs slightly worse at an RMSE of 3.78 kJ mol^−1^ compared to the external test set 1 performance of reference 34 (RMSE 3.20 kJ mol^−1^). However, our final HBD model performs excellently on the HBD test set. For a series of individual HBD to be screened for HBD strength, our final HBD model may therefore be a preferred choice.

### Application example

We now present four molecules that contain both acceptor and donor moieties to illustrate the potential of our ML models (the GPR mean value is reported) and assess the possibility of our models to predict intramolecular HB formation, which often changes the physicochemical properties of molecules, e.g., their solubilities [85].

**Fig. 11:**
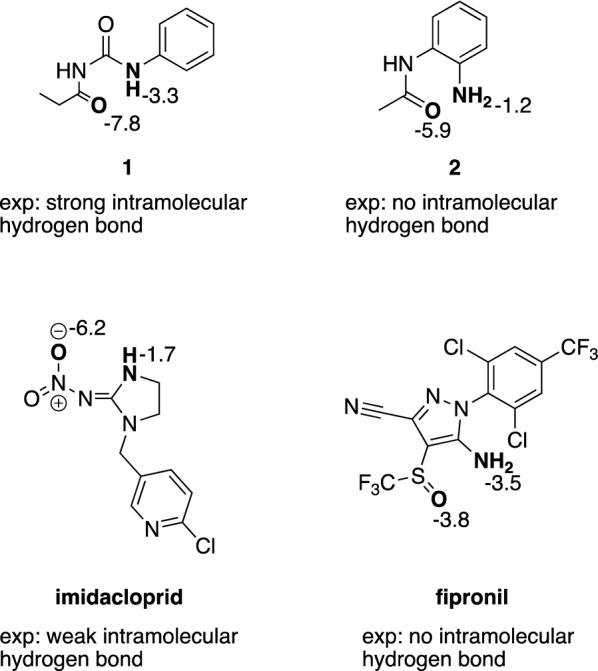
Application example of the trained ML models using the best-performing radial atomic activity descriptors on four molecules. Acceptor and donor atoms that could participate in an internal hydrogen bond are marked in bold and the predicted hydrogen bonding strengths in kJ mol^−1^ for the respective atoms are displayed next to them. The experimental determinations of whether an internal hydrogen bond was formed or not were performed by NMR spectroscopy [86–88]

Figure 11 shows two amides (**1** and **2**) and two agrochemicals, imidacloprid and fipronil. For these compounds, an NMR method [86] has been used to determine the internal hydrogen bond formation. The amide **1** forms a strong intramolecular hydrogen bond whereas the amide **2** does not [87]. The predicted donor strength of the HBD in **1** is − 3.3 kJ mol^−1^ and the predicted acceptor strength for the amide carbonyl HBA is − 7.8 kJ mol^−1^. In **2**, both the acceptor and donor strengths are predicted to be less negative. This is an indication that our quantum-chemically derived ML models for HBA/HBD strengths can explain tendencies in intramolecular HB formation in amides.

The second comparison concerns fipronil (no intramolecular HB formation) and imidacloprid (weak intramolecular HB formation) [88]. In imidacloprid (secondary amine tautomer, which has been detected in the NMR measurement), the HBA strength of the nitro oxygen is predicted to be − 6.2 kJ mol^−1^ and the predicted donor strength of the secondary amine is − 1.7 kJ mol^−1^. Our predicted HBA strength for the sulfinyl of fipronil is − 3.8 kJ mol^−1^ and the predicted HBD strength for the primary amine is − 3.5 kJ mol^−1^. Although the fipronil potential intramolecular HB donor is predicted to be stronger than in imidacloprid, the weaker acceptor may be the cause that no intramolecular HB is formed. Our predicted HBA/HBD strengths are therefore consistent with the experimental determinations of intramolecular HB formation. This indicates the potential of our method’s predicted HBA/HBD strengths to be used as descriptors in a productive setting for molecular design within the context of intramolecular HB formation.

## Summary and conclusions

We presented machine learning models for hydrogen bond acceptor (HBA) and hydrogen bond donor (HBD) strengths, which were trained on quantum chemically computed complexation free energies in solution.

The underlying databases, which are published along with this article, represent a diverse HBA and HBD chemical space and are the largest such databases on record. After a necessary linear fit due to systematic errors of the QC method employed, the RMSE of the computed HBA/HBD strengths are 2.6 kJ mol^−1^ in both cases.

We built ML models on those databases, scanning over both ML models and features using tenfold internal CV. Our previously developed radial atomic descriptors served as the scanned feature space. For ML, we scanned over GPR including different kernel functions and other regression models (linear regression, multilayer perceptron regression, random forest regression, and support vector regression, see Additional file 1) The best-performing final descriptors for HBA and HBD atoms, respectively, each involved a sorted shell descriptor based on CM5 partial charges computed at the GFN-xTB level of theory, and GPR models employing the Matérn kernel. The learning curves derived showed that the variance estimate of the GPR models decreased with growing fractions of training data points, which indicates the usefulness and interpretability of the GPR variance estimate: It could be used as a threshold for an on-the-fly estimation of the models’ applicability domains.

The final mean RMSEs of 4.6 kJ mol^−1^ for the HBA model and 3.8 kJ mol^−1^ for the HBD model in internal CV are far higher than the RMSE of the underlying QC data against experiment and indicate that there is a need for even more data and better performing descriptors in the future. Nevertheless, a striking test set performance of the HBA and HBD final models is obtained with RMSEs of 3.8 kJ mol^−1^ for HBA experimental strengths and 2.3 kJ mol^−1^ for experimental HBD strengths. The donor performance falls within the same range of accuracy of previous models applying quantum chemical descriptors [35] or ISIDA fragment descriptors, trained on experimental HB free energies [38]. QC target values can therefore serve as a full substitute for experiment for HBA/HBD strengths, not only drastically reducing costs compared to experimental determination, but also allowing for the calculation of interaction energies in case of multiple potentially interacting acceptors or donors in one molecule. Finally, it appears that our predicted HBA/HBD strengths could be used as descriptors to classify whether intramolecular H-bond formation will take place or not as the correct trends are observed for the two case studies of provided for one pair of differentially substituted amides and two agrochemicals.

Future work will consist of expanding the openly available databases and to explore the use of novel or different atomic descriptors to improve the internal CV performance of the ML models.

## Additional files

**Additional file 1.** Supporting information (SI), detailing (i) the hydrogen bonding scales used in this study, (ii) generated hydrogen bonding fragments with high frequencies, and (iii) the results of the descriptor scans and additional machine learning regression results.

**Additional file 2.** HBA database.

**Additional file 3.** HBD database.

**Additional file 4.** HBA complexes database.

**Additional file 5.** HBD complexes database.

**Additional file 6.** Documentation for the HBA database.

**Additional file 7.** Documentation for the HBD database.

**Additional file 8.** Source code for training the HBA and HBD models and two example molecules.

## Data Availability

Four databases in.sdf format are submitted as additional files with this article. These are the acceptor (Additional file 2) and donor databases (Additional file 3) with their 2D structures and the optimized complex coordinates with their 3D structures (Additional files 4, 5). All the molecular properties are documented in Additional files 6 and 7 (pdf). Source code to train the acceptor and donor models and two test molecules are provided in Additional file 8 (zipped archive).
