# Supplementary material for: Machine learning models for hydrogen bond donor and acceptor strengths using large and diverse training data generated by first-principles interaction free energies
Source: J Cheminform. 2019 Sep 11;11:59. doi: 10.1186/s13321-019-0381-4 (PMC6737620; doi:10.1186/s13321-019-0381-4)
Supplement: Supplementary file 1 — Additional file 1. Supporting information (SI), detailing (i) the hydrogen bonding scales used in this study, (ii) generated hydrogen bonding fragments with high frequencies, and (iii) the results of the descriptor scans and additional machine learning regression results. [file 13321_2019_381_MOESM1_ESM.docx]

Machine learning models for hydrogen bond donor and acceptor strengths using large and diverse training data generated by first-principles interaction free energies

Christoph A. Bauer,^1^ Gisbert Schneider,^1^ Andreas H. Göller ^2^*

[1] Swiss Federal Institute of Technology (ETH), Department of Chemistry and Applied Biosciences, 8093 Zurich (Switzerland)

[2] Bayer AG, Pharmaceuticals, R&D, 42096 Wuppertal (Germany)

SUPPORTING INFORMATION

**Summary**

Figure S1 shows the two hydrogen bonding scales used in this study with their respective reference donor/acceptor molecules 4-fluorophenol and acetone.

Table S1 shows the hydrogen bonding acceptor/donor fragments with the highest incidence generated from the 276,004 ChEMBL23 active compounds. Table S2 shows high-incidence donor fragments generated from the same set.

The descriptor and kernel function scan results for the training of the Gaussian Process Regression models are presented in Tables S3 and S5 for the hydrogen bond acceptors and donors, respectively. The final combinatorial descriptor/model combination is described for the hydrogen bond acceptors and donors, respectively. Additional machine learning models and their results are presented in Tables S4 and S6. The HBA and HBD test set target value distributions are found in Figure S2.

**Hydrogen Bonding Scales Used in This Study**

Figure S1: The two HB scales (acceptors and donors) used in this work. The reference donor is 4-fluorophenol and the reference acceptor is acetone.

**High-Incidence Fragments Generated from the ChEMBL23 actives data set**

Table S 1 Top 10 hydrogen bond acceptor fragments by incidence, generated from 276,004 ChEMBLl23 actives.

| Fragment structure | Incidence | Acceptor(s) | Function(s) |
| --- | --- | --- | --- |
|  | 5,882 | Oxygen(s) | Carboxylic acid |
|  | 4,426 | Oxygen, nitrogen | Morpholine |
|  | 3,825 | Oxygen(s), nitrogen | Sulfonamide |
|  | 3,204 | Oxygen(s), nitrogen | alpha-amino carboxylic acid |
|  | 2,744 | Oxygen(s) | Nitro |
|  | 2,708 | Oxygen(s) | Benzoic acid |
|  | 2,587 | Nitrogen | Benzonitrile |
|  | 2,343 | Nitrogen | Pyridine |
|  | 1,874 | Oxygen(s) | beta-oxy carboxylic acid |
|  | 1,788 | Oxygen(s) | Sulfone |

Table S 2: Selection of frequent hydrogen bond donor fragments and their incidence, generated from 276,004 ChEMBL23 actives.

| Fragment structure | Incidence | Donor atom | Function |
| --- | --- | --- | --- |
|  | 2,941 | Oxygen | Carboxylic acid |
|  | 1,354 | Oxygen | Benzoic acid |
|  | 1,323 | Oxygen | Phenol |
|  | 1,275 | Nitrogen | Sulfonamide |
|  | 946 | Nitrogen | Piperidine |
|  | 330 | Carbon | Alkyne |
| **** | 154 | Sulfur | Thiol |

**Hydrogen Bond Acceptor Database Machine Learning Models: Additional Information**

Table S 3 Descriptors and their parameters + kernels in Gaussian Process Regression scanned for training the Gaussian Process Regression Model on the HB acceptors database. The optimal property types (where there are more than one) and parameters are marked in **bold**.

| Descriptor | Property Type | Parameter | Range | Kernel^a^ |
| --- | --- | --- | --- | --- |
| Charge Shell | Mulliken, **CM5** | Number of shells | **3**,4,5,6,7 | M0.5, **M1.5,** M2.5, RBF, RQ |
| Mass Shell | Atomic masses | Number of shells | **3**,4 | M0.5, **M1.5**, M2.5, RBF, RQ |
| Sorted Shells (CIP Sort = True) | Mulliken, **CM5** | Number of shells | 2,3,**4** | M0.5, **M1.5**, M2.5, RBF,RQ |
| Autocorrelation function (3D, rmin=1.0) | Mulliken, **CM5** | rmax  step size | **6.0**, 8.0, 10.0  **0.2**, 0.5 | M0.5, **M1.5**, M2.5, RBF, RQ |
| Autocorrelation function (topological) | Mulliken, **CM5** | rmax | **3**,4,5 | M0.5, **M1.5**, M2.5, RBF, RQ |
| Radial distribution function (3D, rmin= 1.0) | Mulliken, **CM5** | rmax  step size | 6.0, 8.0, **10.0**  **0.2**, 0.5 | M0.5, **M1.5**, M2.5, RBF,RQ |

^a^Gaussian process regression kernel function abbreviations: M=Matérn 0.5,1.5 and 2.5 were the scanned values of *v*, RBF=radial basis function, RQ=rational quadratic.

**Best combinatorial descriptor/ GPR kernel description for the acceptor database**

The final combinatorial descriptor had 151 dimensions and the parameters: Sorted shell descriptor (CM5, number of shells = 3), autocorrelation function (3D, CM5, rmax = 10.0, step size = 0.2), charge shell (CM5, number of shells = 7). This parameter combination was found in an additional grid search for the combination of descriptor types. The final kernel used in GPR was the combined Matern *v*=1.5 kernel.

**ML model scan using two descriptor types for the acceptor database**

Two descriptors, one medium-performing and the final combinatorial descriptor, were tested in combination with different machine learning models as implemented in scikit-learn 0.19.1. The default parameters of those model types were used unless specified otherwise. The models were trained on the full quantum chemical acceptor database. The results are judged by the performance on the experimental test set, see Table S2.

Table S 4: Performance on experimental test set (917 compounds from the pK_BHX_ database) using two different descriptor types and various machine learning models trained on the acceptor database. LR = linear regression; RF = random forest regression; MLP (500 hidden) = multilinear perceptron regression with 500 hidden layers; SVR (lin) = support vector regression with a linear kernel; GPR (M1.5) = Gaussian Process Regression with a Matérn kernel (v=1.5). The final model (presented also in the main manuscript) is marked **in bold.**

| Metric | LR | RF | MLP  (500 hidden) | SVR  (lin) | GPR (M1.5) |
| --- | --- | --- | --- | --- | --- |
| Sorted shell descriptor (CM5, CIP sorted, 3 shells) 53 dimensions | | | | | |
| R^2^ | 0.41 | 0.24 | 0.46 | 0.25 | 0.48 |
| RMSE (kJ mol^-1^) | 4.28 | 4.87 | 4.50 | 4.83 | 4.04 |
| Spearman R | 0.70 | 0.53 | 0.75 | 0.60 | -- |
| Final combinatorial descriptor (151 dimensions) | | | | | |
| R^2^ | 0.37 | 0.10 | 0.49 | 0.22 | **0.54** |
| RMSE (kJ mol^-1^) | 4.44 | 5.31 | 3.98 | 4.94 | **3.78** |
| Spearman R | 0.67 | 0.38 | 0.75 | 0.52 | **0.77** |

**Hydrogen Bond Donor Database Machine Learning Models: Additional Information**

Table S 5 Descriptors and their parameters + kernels in Gaussian Process Regression scanned for training the Gaussian Process Regression Model on the HB donors database. The optimal property types (where there are more than one) and parameters are marked in **bold**.

| Descriptor | Property Type | Parameter | Range | Kernel^a^ |
| --- | --- | --- | --- | --- |
| Charge Shell | Mulliken, **CM5** | Number of shells | **3**,4,5,6,7 | **M0.5**, M1.5, M2.5, RBF, RQ |
| Mass Shell | Atomic masses | Number of shells | **2**,3,4 | **M0.5**, M1.5, M2.5, RBF, RQ |
| Sorted Shells (CIP Sort = True) | Mulliken, **CM5** | Number of shells | **2**,3,4 | **M0.5**, M1.5, M2.5, RBF, RQ |
| Autocorrelation function (3D, rmin=1.0) | Mulliken, **CM5** | rmax  step size | **6.0**, 8.0, 10.0  0.2, **0.5** | **M0.5**, M1.5, M2.5, RBF, RQ |
| Autocorrelation function (topological) | Mulliken, **CM5** | rmax | **3**,4,5 | **M0.5**, M1.5, M2.5, RBF, RQ |
| Radial distribution function (3D, rmin= 1.0) | Mulliken, **CM5** | rmax  step size | 6.0, **8.0**, 10.0  **0.2**, 0.5 | **M0.5**, M1.5, M2.5, RBF, RQ |

^a^Gaussian process regression kernel function abbreviations: M=Matérn 0.5,1.5 and 2.5 were the scanned values of *v*, RBF=radial basis function, RQ=rational quadratic.

**Best combinatorial descriptor/ GPR kernel description for the donor database**

The final combinatorial descriptor had 115 dimensions. The parameters were: Sorted shell descriptor (CM5, number of shells = 2), autocorrelation function (3D, CM5, rmax = 10.0, step size = 0.2), charge shell (CM5, number of shells = 7). This parameter combination was found in an additional grid search for the combination of descriptor types. The final used kernel in GPR was the combined Matern *v*=0.5 kernel.

**ML model scan using two descriptor types for the donor database**

Two descriptors, one medium-performing and the final combinatorial descriptor, were tested in combination with different machine learning models as implemented in scikit-learn 0.19.1. The default parameters of those model types were used unless specified otherwise. The models were trained on the full quantum chemical donor database. The results are judged by the performance on the experimental test set, see Table S2.

Table S 6 Performance on the experimental test set (58 compounds from the Strasbourg database) using two different descriptor types and various machine learning models trained on the acceptor database. LR = linear regression; RF = random forest regression; MLP (500 hidden) = multilinear perceptron regression with 500 hidden layers; SVR (lin) = support vector regression with a linear kernel; GPR (M0.5) = Gaussian Process Regression with a Matérn kernel (v=0.5). The final model (presented also in the main manuscript) is marked **in bold.**

| Performance on experimental test set (58 compounds) | LR | RF | MLP (500 hidden) | SVR (lin) | GPR (M0.5) |
| --- | --- | --- | --- | --- | --- |
| Sorted shell descriptor (CM5, CIP sorted, 2 shells) 17 dimensions | | | | | |
| R^2^ | -0.27 | 0.43 | 0.34 | -0.41 | 0.56 |
| RMSE (kJ mol^-1^) | 5.17 | 3.45 | 3.73 | 5.07 | 3.04 |
| Spearman R | 0.04 | 0.64 | 0.59 | -0.05 | -- |
| Final combinatorial descriptor (115 dimensions) | | | | | |
| R^2^ | 0.15 | 0.43 | 0.67 | -0.26 | **0.74** |
| RMSE (kJ mol^-1^) | 4.24 | 3.46 | 2.63 | 5.14 | **2.34** |
| Spearman R | 0.67 | 0.64 | 0.80 | 0.14 | **0.88** |

**Target value distributions of the HBA and HBD experimental test sets**


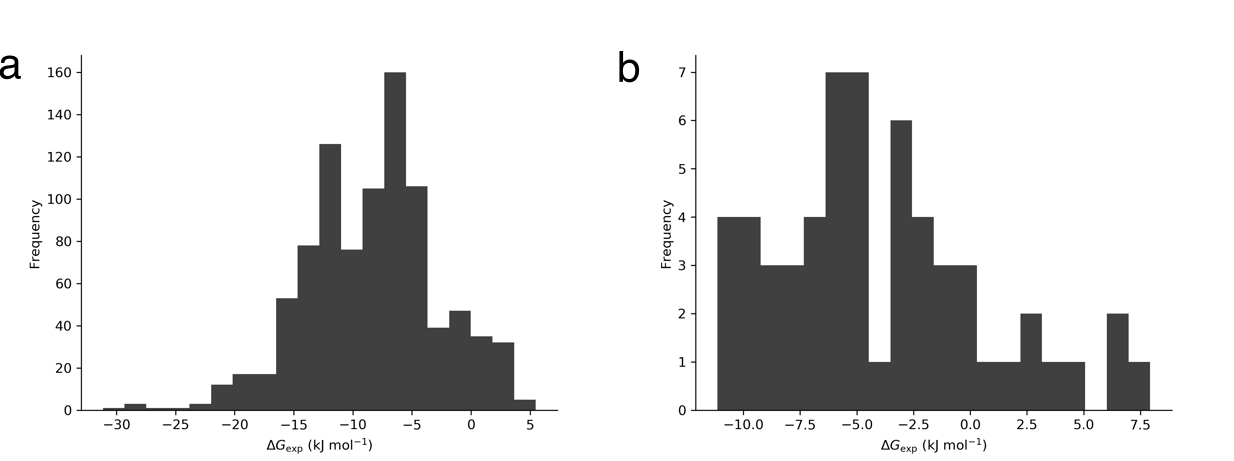


FigureS2: Test set experimental target value distributions of HBA strengths (a), and HBD strengths (b)
