# Supplementary material for: Machine learning models for hydrogen bond donor and acceptor strengths using large and diverse training data generated by first-principles interaction free energies
Source: J Cheminform. 2019 Sep 11;11:59. doi: 10.1186/s13321-019-0381-4 (PMC6737620; doi:10.1186/s13321-019-0381-4)
Supplement: Supplementary file 6 — Additional file 6. Documentation for the HBA database. [file 13321_2019_381_MOESM6_ESM.pdf]

The QC Hydrogen Bond Acceptor Database Documentation

Contents

1 Structures and Methods **2**
1.1 Fragment Generation and Selection 2
1.2 Quantum Chemical Method 3

2 Properties **4**
2.1 E_el 4
2.2 HBA_atom_index 4
2.3 Function 5
2.4 Atom 5
2.5 Mol_ID 5
2.6 E_el_PW6B95 5
2.7 E_el_PBEh-3c 5
2.8 E_el complex_PW6B95 5
2.9 E_el complex_PBEh-3c 5
2.10 HB_distance 6
2.11 dG(298)_RRHO (kJ/mol) 6
2.12 dG(298)_RRHO complex (kJ/mol) 6
2.13 dG_solv_SMD_CCl4 (kJ/mol) 6
2.14 dG_solv_SMD_CCl4 complex (kJ/mol) 6
2.15 pKBHX_QC 6
2.16 Delta G_sol (kJ/mol) 7
2.17 Delta G (kcal/mol) 7
2.18 3D Complex Structures 8

3 Statistics **8**
3.1 Distributions of Target Values (kJ mol^{-1}) 8
3.2 Distributions of HB Distances 19

1 Structures and Methods

The QC hydrogen bond acceptor database holds quantum chemically computed hydrogen bond acceptor strengths, assigned to hydrogen bond acceptor (HBA) atoms. 3,039 2D molecular structures are stored in the database. They originate from 276,004 ChEMBL23 active compounds and represent acceptor moieties. Each structure may have one to four acceptor sites. The acceptor atoms are oxygens and nitrogens.

1.1 Fragment Generation and Selection

The strategy to generate the fragment structures is as follows:

1. Define hydrogen bond acceptor sites: Every oxygen, every nitrogen.
2. Iterate over all acceptor sites: Get the atom environment (substructure) up to the 4th shell. Three cases are defined then:
   (a) Chain fragment: Atoms around the HBA site are not in any ring up to the third shell. If fourth shell atoms are in a ring, the atom type is changed to an sp^3 atom.
   (b) Ring + side chain fragment: At least one oatom within the third shell around the BHA site is part of a ring. The whole ring is taken in addition to the side chain, which extends to the fourth shell.
   (c) Ring fragment: The HBA site is in a ring. The whole rings system and any side chains up to the fourth shell are taken.

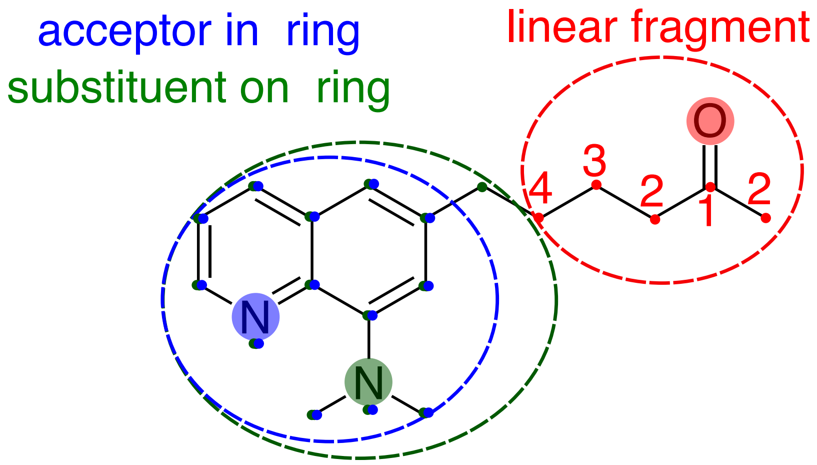

Figure 1: Fragmentation strategy to arrive at the molecular structures

162,731 unique fragments are thus generated. Structures are discarded (filtered) by the following criteria:

- all inorganic structures
- all phosphorous containing structures
- all structures with a corrected molecular weight (MW_{corr} = MW with all halogen atoms given the mass of flourine atoms) > 300.
- all structures with more than three rings

. Quantum chemical calculations were performed on the selected structures and on generated complexes with the reference hydrogen bond donor para-fluorophenol. 3,118 structures had at least one complete set of successful quantum chemical computations, and thus at least one computed pK_{BHX} value.

1.2 Quantum Chemical Method

The computation of hydrogen bond acceptor strengths by quantum chemistry consisted of the following protocol:

Protocol for generating 3D structures of the acceptor molecules and 3D structures of the complexes with para-fluorophenol as the hydrogen bond donor:

1. Add hydrogens to the 2D structure by the rdkit, Version 2017.09.1. The tautomer structure is determined by the bond block. The bond orders were taken in the kekulized form as provided by the original ChEMBL23 actives data set.
2. Generate one 3D structure (conformer) by the ETKDG method as implemented by Riniker and Landrum in the rdkit, Version 2017.09.1.
3. Find hydrogen bond acceptor sites.
4. Compute the electronic structure at the GFN-xTB level of theory and get the charge centers of the Foster-Boys localized molecular orbitals (lone pairs) associated with the acceptor sites.
5. Place the donated hydrogen of para-fluorophenol at 2 Ådistance from the charge center of the lone pair at an angle of 180°.
6. Optimize the structure for 100 steps using the MMFF94s force field as implemented in the rdkit, Version 2017.09.1 by Landrum. The hydrogen bond distance is constrained to 2 Å. The hydrogen bond angle is constrained to 180°.

Protocol for computing the hydrogen bond acceptor strength quantum chemically:

1. Optimize the 3D structures of the acceptor molecules and the complexes at the PBEh-3c level of theory using TURBOMOLE, version 7.0.2.
2. Compute the harmonic frequencies of the acceptor molecules and the complexes at the PBEh-3c level of theory using TURBOMOLE, version 7.0.2.
3. Compute the single point energies of the acceptor molecules and the complexes at the PW6B95-D3(BJ)/def2-QZVP level of theory using TURBOMOLE, version 7.0.2.
4. Compute the solvation free energies at the SMD(BP86/def2-TZVP) level of theory for the solvent CCl_4 using Gaussian 09.

All the quantum chemically determined energies for the acceptor molecules and their complexes are reported in the database. The energies for the hydrogen bond donor molecule para-fluorophenol are as follows:

Table 1: Energies of para-fluorophenol optimized at the PBEh-3c level of theory.

	energy
E (PW6B95-D3(BJ)/def2-QZVP)	-407.3936234012 E_h
G (RRHO correction)	182.67 kJ mol^{-1}
δG_solv (CCl_4)	-23.68 kJ mol^{-1}

2 Properties

All properties contained by the molecules in the SDF file are described in this section.

2.1 E_el

This is the electronic energy at the PBEh-3c level of theory

2.2 HBA_atom_index

These are the indices of the HBA atoms. There is a new line character after every index if there is more than one acceptor site in the structure.

2.3 Function

These are the functional groups as determined by rdkit substructre matching. Statistics about the functional groups are found in section 3. There is a new line character after every functional group if there is more than one acceptor site in the structure. If the acceptor function could not be determined by the algorithm, 'undefined acceptor function' is placed.

2.4 Atom

This can be 'N' or 'O'. If there is more than one acceptor site in the structure, they are separated by new line characters.

2.5 Mol_ID

This is the identifier. It indicates which ChEMBL molecule is the origin of the fragment.

2.6 E_el_PW6B95

This is the electronic energy at the PW6B95-D3(BJ)/def2-QZVP level of theory for the structure optimized at the PBEh-3c level of theory. This value is reported in E_{h}.

2.7 E_el_PBEh-3c

This is the electronic energy at the PBEh-3c level of theory for the structure optimized at the PBEh-3c level of theory. This value is reported in E_{h}.

2.8 E_el complex_PW6B95

This is the electronic energy at the PW6B95-D3(BJ)/def2-QZVP level of theory for the complex with para-fluorophenol optimized at the PBEh-3c level of theory. This value is reported in E_{h}. If there is more than one complex, one value is reported for each complex, separated by new line characters.

2.9 E_el complex_PBEh-3c

This is the electronic energy at the PW6B95-D3(BJ)/def2-QZVP level of theory for the complex with para-fluorophenol optimized at the PBEh-3c level of theory. This value is reported in E_{h}. If there is more than one complex, one value is reported for each complex, separated by new line characters.

2.10 HB_distance

This is the hydrogen bond distance of the 3D complex structure with para-fluorophenol as the hydrogen bond donor in Å, optimized at the PBEh-3c level of theory.

2.11 dG(298)_RRHO (kJ/mol)

This is the thermal correction to the free energy in the gas phase as computed by the rigid rotor harmonic oscillator approximation for the structure optimized at the PBEh-3c level of theory.

2.12 dG(298)_RRHO complex (kJ/mol)

This is the thermal correction to the free energy in the gas phase as computed by the rigid rotor harmonic oscillator approximation for the complex with para-fluorophenol optimized at the PBEh-3c level of theory. If there is more than one complex, one value is reported for each complex, separated by new line characters.

2.13 dG_solv_SMD_CCl4 (kJ/mol)

This is the solvation free energy as computed by the SMD(BP86/def2-TZVP) level of theory for the structure optimized at the PBEh-3c level of theory.

2.14 dG_solv_SMD_CCl4 complex (kJ/mol)

This is the solvation free energy as computed by the SMD(BP86/def2-TZVP) level of theory for the complex with para-fluorophenol optimized at the PBEh-3c level of theory. If there is more than one complex, one value is reported for each complex, separated by new line characters.

2.15 pKBHX_QC

This is the computed acceptor strength, computed from the following regression formula:

$$\mathrm{p}K_{\mathrm{BHX\_QC}} = (0.56 \times \Delta G_{\mathrm{sol}} - 20.12 \mathrm{kJ\ mol^{-1}})/ - 5.705 \quad (1)$$

If the computation was not successful, it becomes 'no computed pKBHX value!'. This allows for entries in the database where at least one computation was successful, but not all.

2.16 Delta G_sol (kJ/mol)

This is the computed ΔG_{sol} value as given by the formula:

$$\Delta G_{\text{sol}} = G_{\text{sol}}(\text{complex}) - G_{\text{sol}}(\text{para} - \text{fluorophenol}) - G_{\text{sol}}(\text{acceptormolecule}) \quad (2)$$

If there is more than one complex, one value is reported for each complex, separated by new line characters.

2.17 Delta G (kcal/mol)

This value is computed by multiplying $\text{p}K_{\text{BHX\_QC}}$ by 1.364.

2.18 3D Complex Structures

A 3D sdf database is supplied with each 3D complex structure (PBEh-3c optimized coordinates) supplied. All properties above are copied to the 3D sdf accordingly, i.e., the first complex gets the first target value etc. Only the entries with successful QC computations are found in the 3D sdf.

3 Statistics

This section summarizes the data according to the functional groups involved. The means (μ) for the hydrogen bond distances (Å) and target values (HBA strengths, kJ mol^{-1}) are shown in Table 2.

Table 2: Acceptor database analysis by functional groups. .

function	atom	data points	μ(HB distance)/Å	$\mu(\Delta G)$/kJ mol^{-1}
alcohol	O	433	1.95	-4.27
aromatic ether	O	13	2.02	-3.38
azide	N	4	2.04	-2.65
carbonyl	O	1382	1.86	-8.18
ether	O	185	1.87	-5.88
imidazole	N	119	1.83	-11.54
imine	N	138	1.81	-13.05
nitrate	O	2	2.28	2.40
nitrile	N	87	1.99	-5.72
phenol ether	O	166	2.04	-2.78
pyrazole	N	103	1.88	-9.16
pyridine	N	298	1.85	-10.66
pyrimidine	N	173	1.86	-8.28
sec. amine	N	387	1.80	-10.02
sulfinyl	O	451	1.95	-5.61
tertiary amine	N	355	1.88	-7.88
undefined acc.	N/O	130	1.93	-6.90
total	N/O	4426	1.89	-7.72

3.1 Distributions of Target Values (kJ mol^{-1})

The distribution of all HBA strengths (ΔG for para-fluorophenol complex formation) is shown in Fig. 20

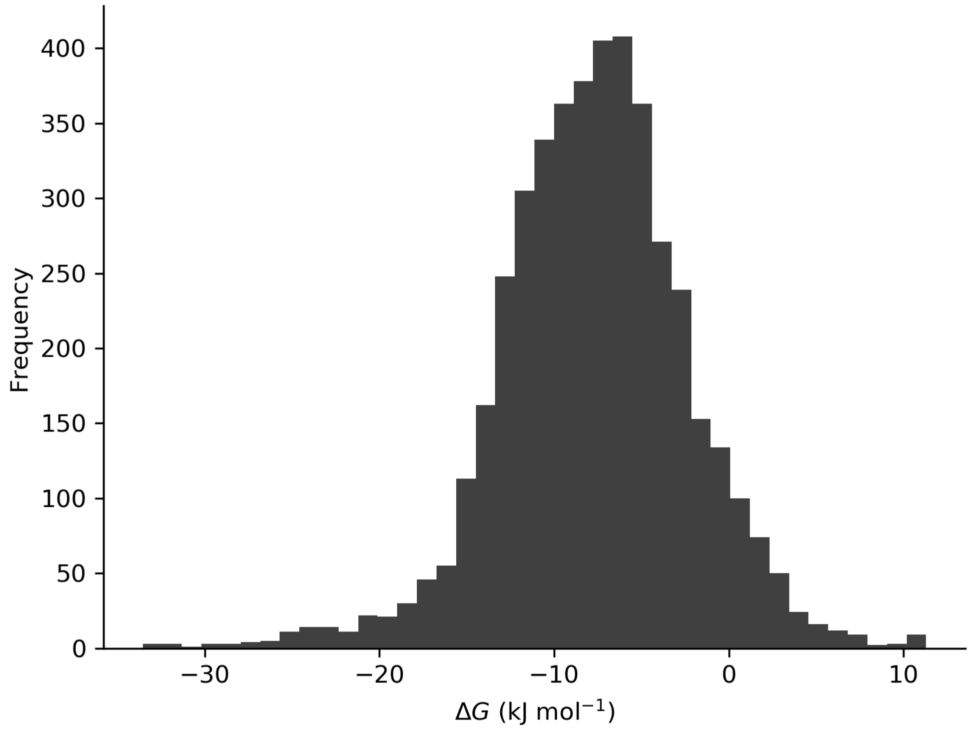

Figure 2: HBA strengths (ΔG for para-fluorophenol complex formation) for the total pKBHX database (4426 data points).

The following figures contain the distributions of target values by functional groups within the database.

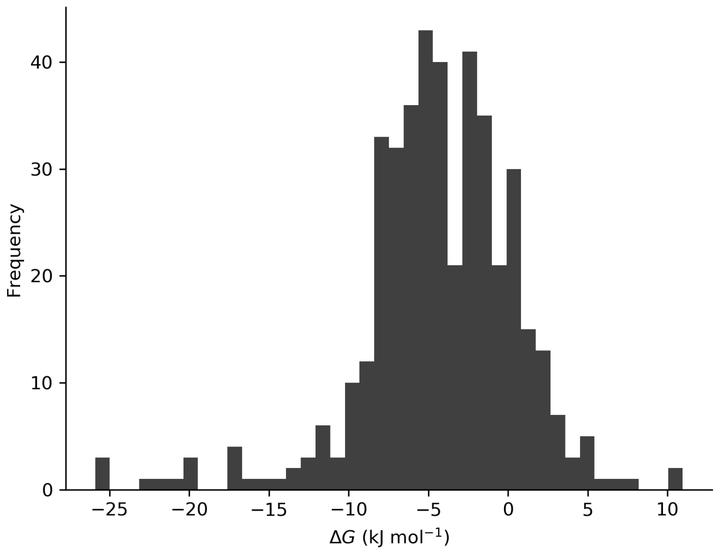

Figure 3: HBA strengths (ΔG for para-fluorophenol complex formation) for alcohols (433 data points).

ether-targetvalue-distribution-kJoules.png

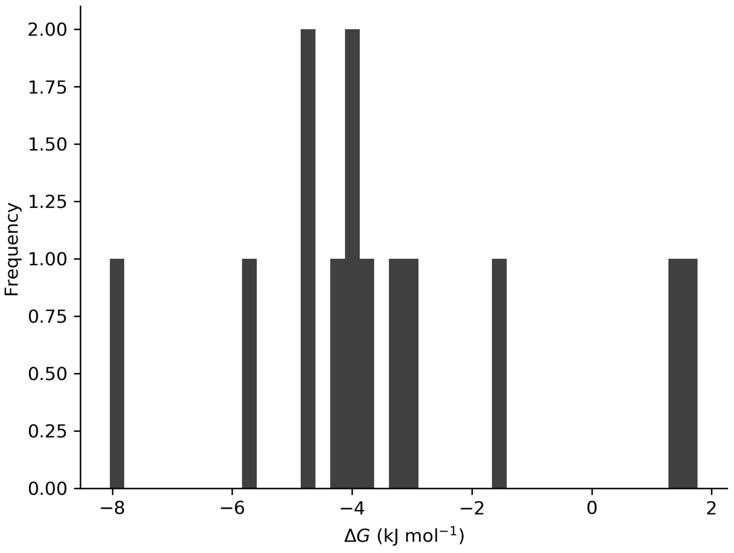

Figure 4: HBA strengths (ΔG for para-fluorophenol complex formation) for aromatic ethers (13 data points).

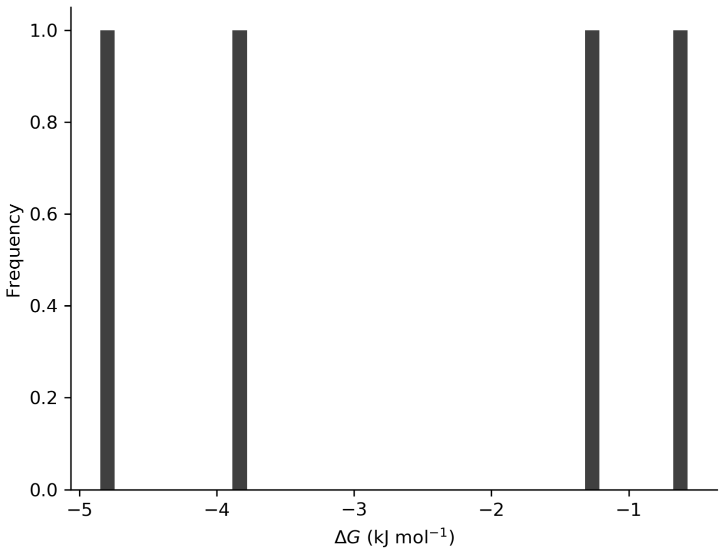

Figure 5: HBA strengths (ΔG for para-fluorophenol complex formation) for azides (4 data points).

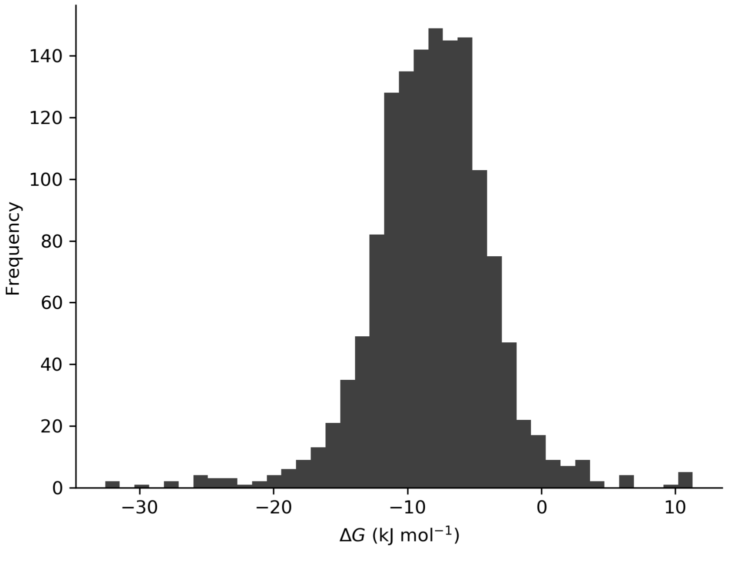

Figure 6: HBA strengths (ΔG for para-fluorophenol complex formation) for carbonyls (1382 data points).

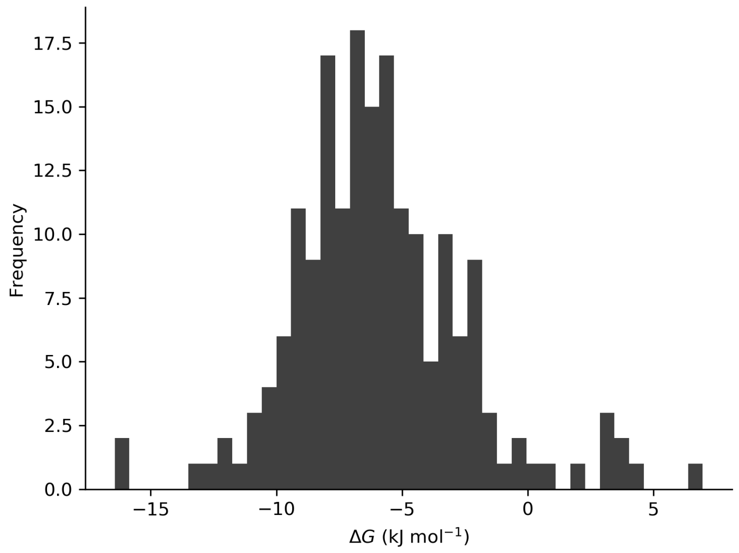

Figure 7: HBA strengths (ΔG for para-fluorophenol complex formation) for ethers (185 data points).

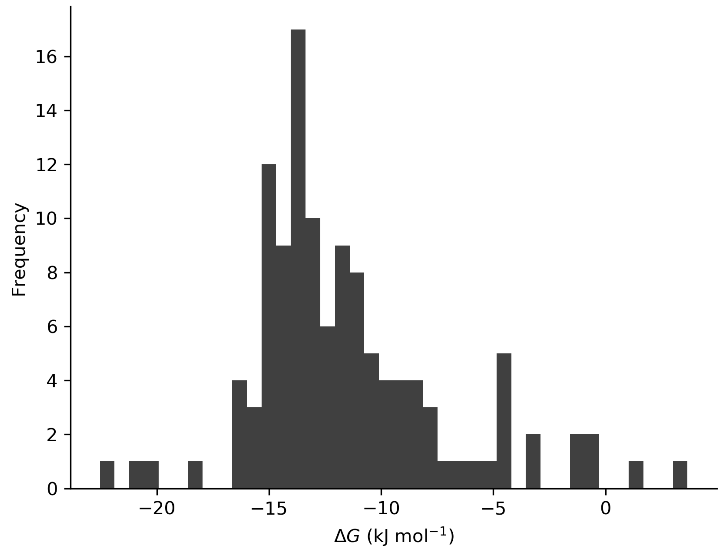

Figure 8: HBA strengths (ΔG for para-fluorophenol complex formation) for imidazoles (119 data points).

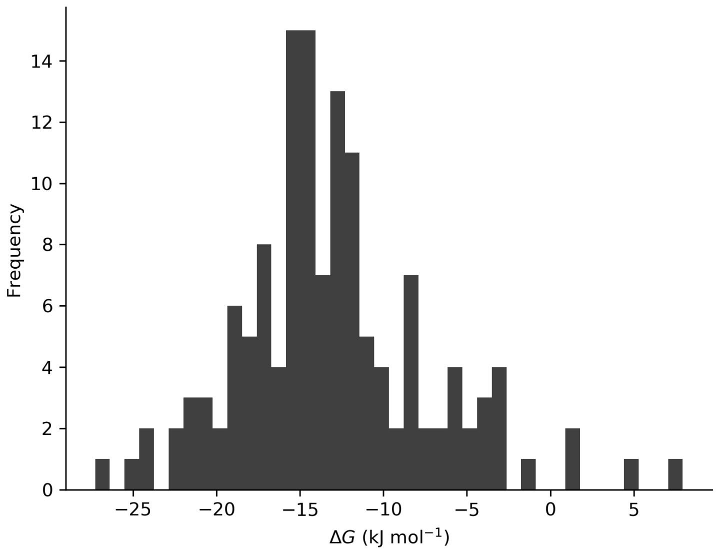

Figure 9: HBA strengths (ΔG for para-fluorophenol complex formation) for imines (138 data points).

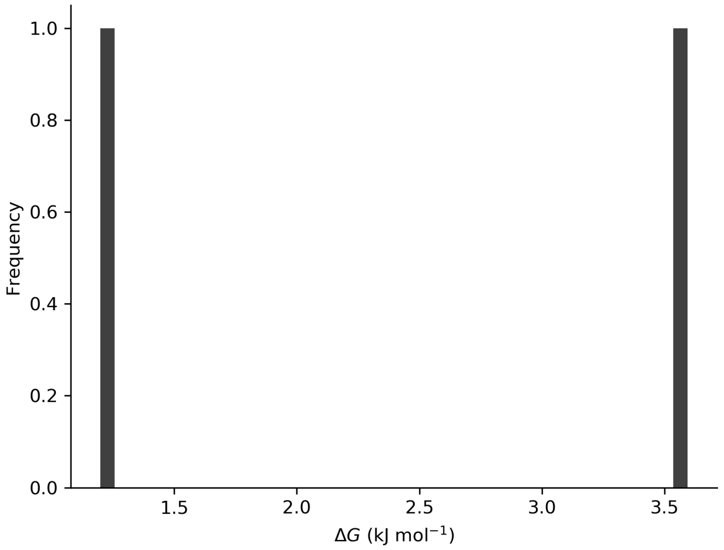

Figure 10: HBA strengths (ΔG for para-fluorophenol complex formation) for nitrates (2 data points).

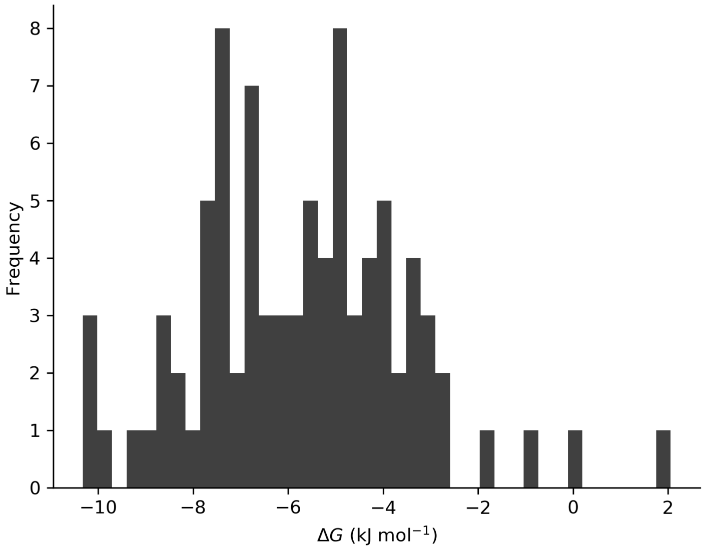

Figure 11: HBA strengths (ΔG for para-fluorophenol complex formation) for nitriles (87 data points).

ether-targetvalue-distribution-kJoules.png

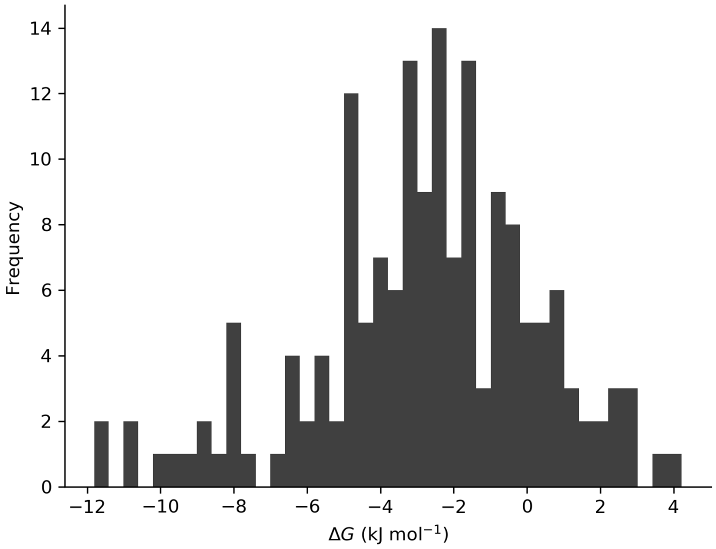

Figure 12: HBA strengths (ΔG for para-fluorophenol complex formation) for phenol ethers (166 data points).

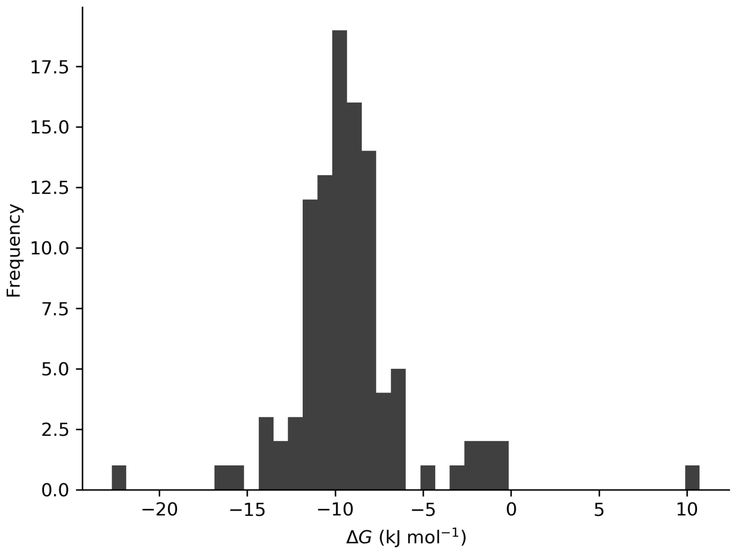

Figure 13: HBA strengths (ΔG for para-fluorophenol complex formation) for pyrazoles (105 data points)103.

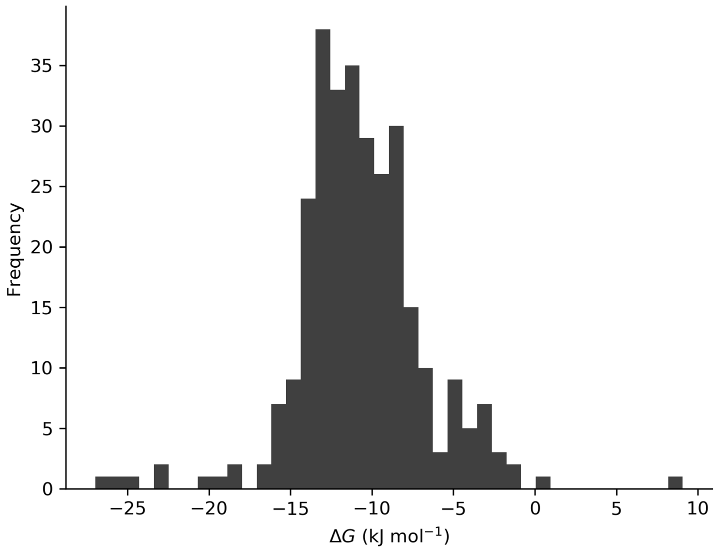

Figure 14: HBA strengths (ΔG for para-fluorophenol complex formation) for pyridines (298 data points).

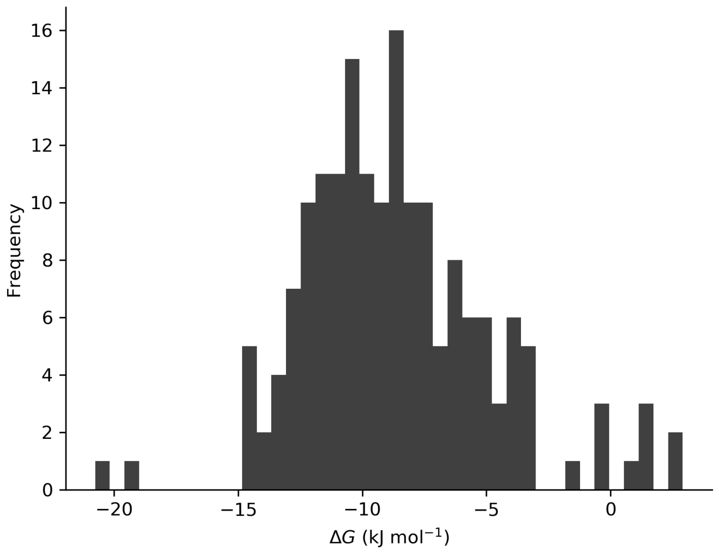

Figure 15: HBA strengths (ΔG for para-fluorophenol complex formation) for pyrimidines (173 data points).

amine-targetvalue-distribution-kJoules.png

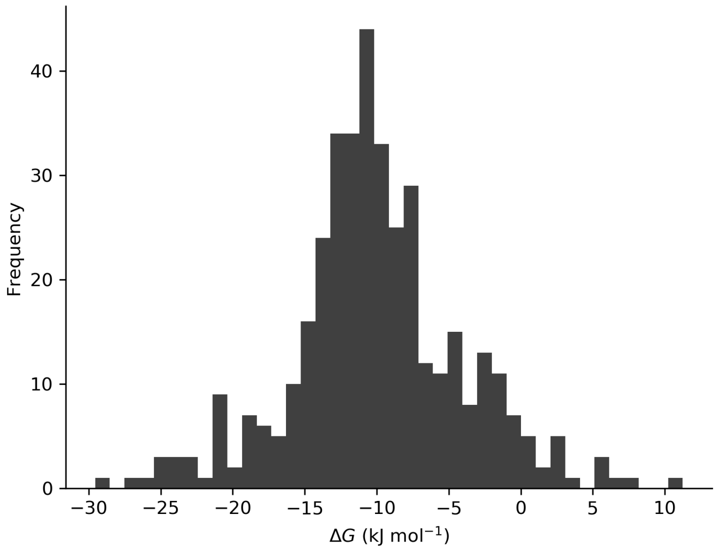

Figure 16: HBA strengths (ΔG for para-fluorophenol complex formation) for secondary amines (387 data points).

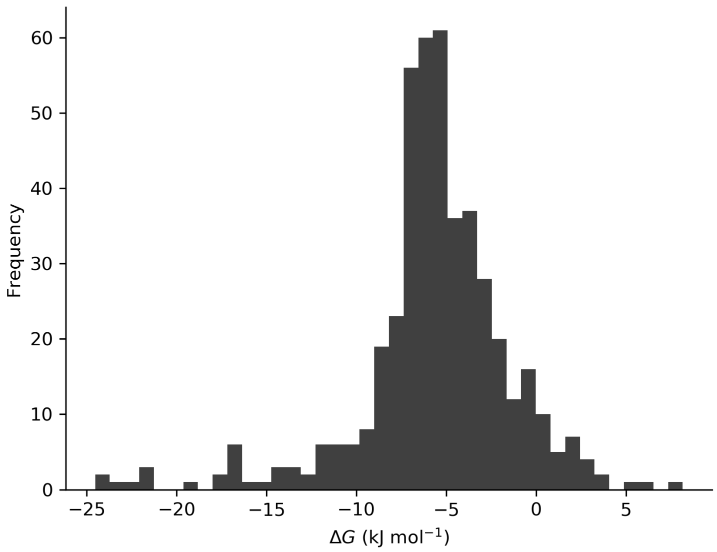

Figure 17: HBA strengths (ΔG for para-fluorophenol complex formation) for sulfinyls (451 data points).

amine-targetvalue-distribution-kJoules.png

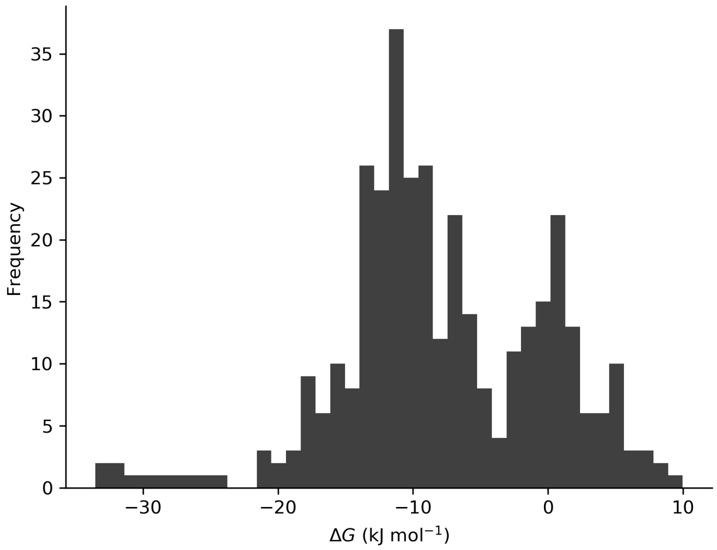

Figure 18: HBA strengths (ΔG for para-fluorophenol complex formation) for tertiary amines (355 data points).

acceptor function-targetvalue-distribution-kJoules.png

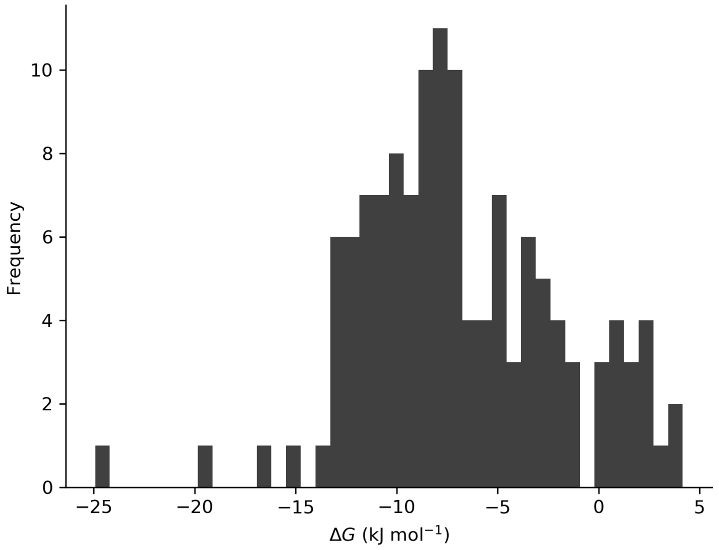

Figure 19: HBA strengths (ΔG for para-fluorophenol complex formation) for undefined acceptor functions (130 data points).

3.2 Distributions of HB Distances

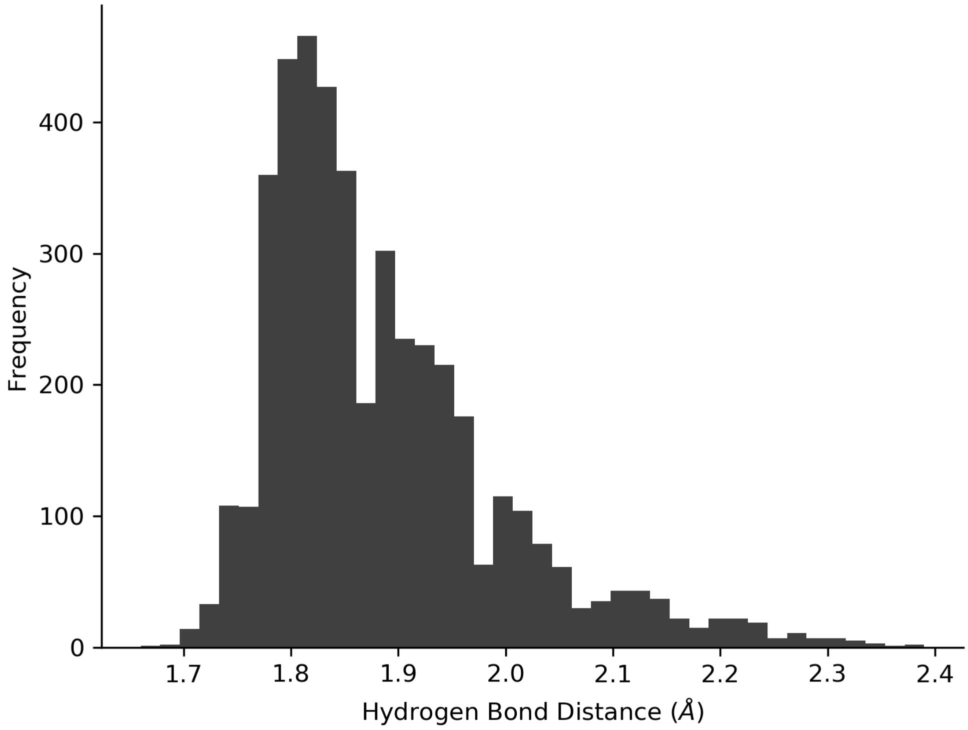

Figure 20: Hydrogen bond distance distribution (Å) for the total QC hydrogen bond acceptor database (4426 data points).

The following figures contain the distributions of hydrogen bond distances by functional groups within the database.

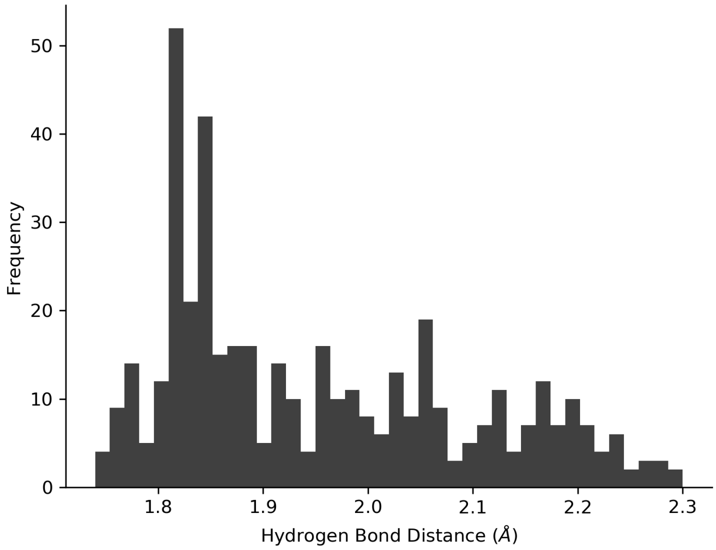

Figure 21: Hydrogen bond distance distribution (Å) for alcohols (433 data points).

ether-HBdistance-distribution.png

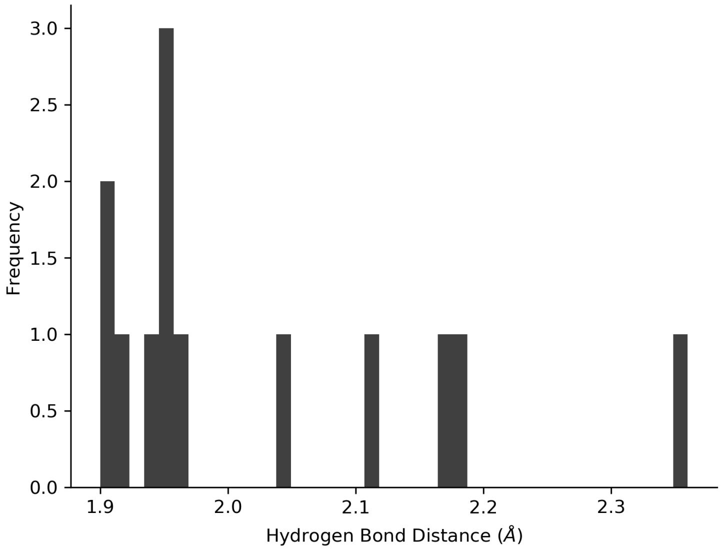

Figure 22: Hydrogen bond distance distribution (Å) for aromatic ethers (13 data points).

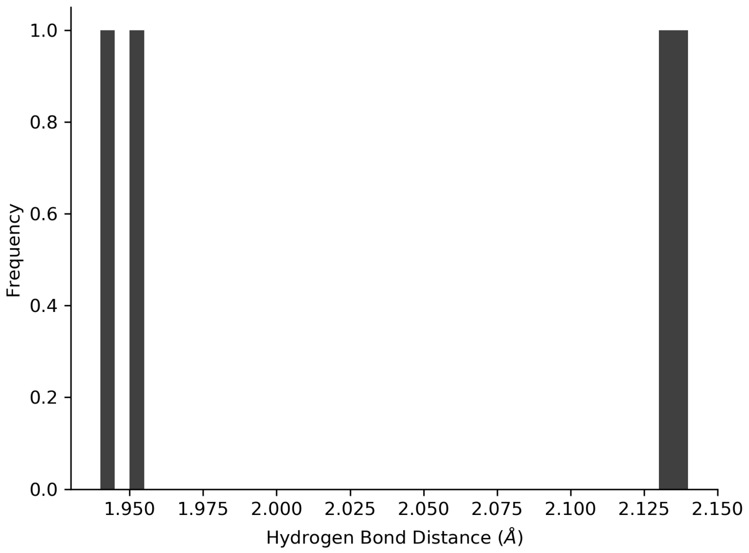

Figure 23: Hydrogen bond distance distribution (Å) for azides (4 data points).

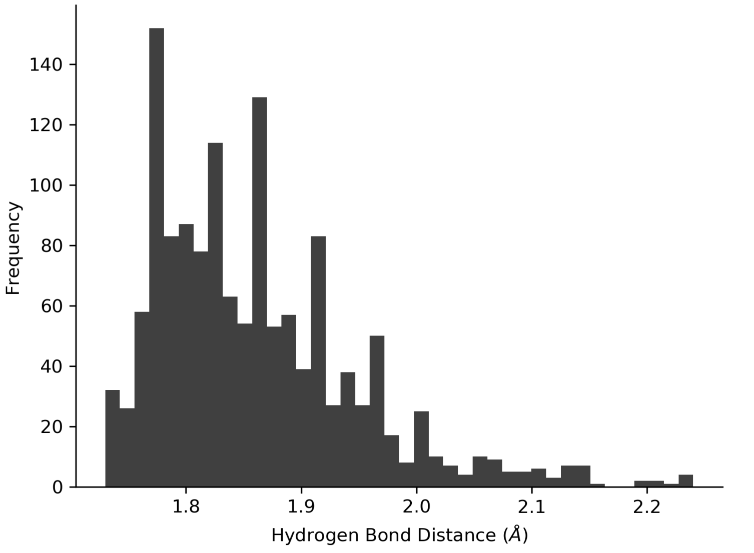

Figure 24: Hydrogen bond distance distribution (Å) for carbonyls (1382 data points).

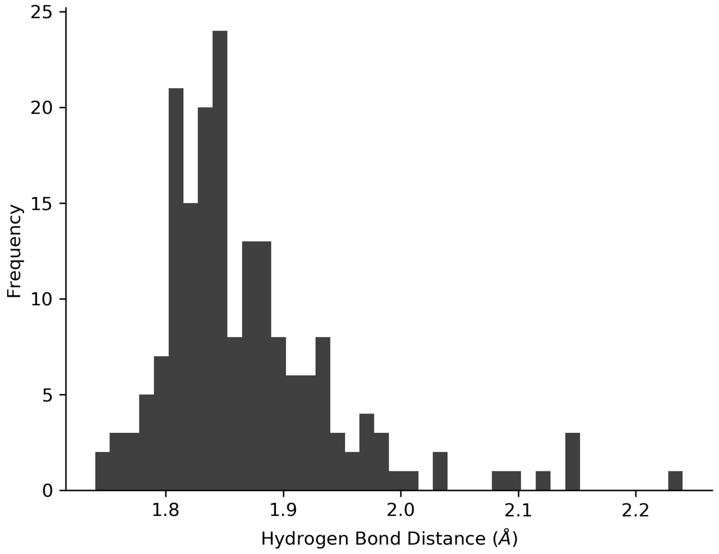

Figure 25: Hydrogen bond distance distribution (Å) for ethers (185 data points).

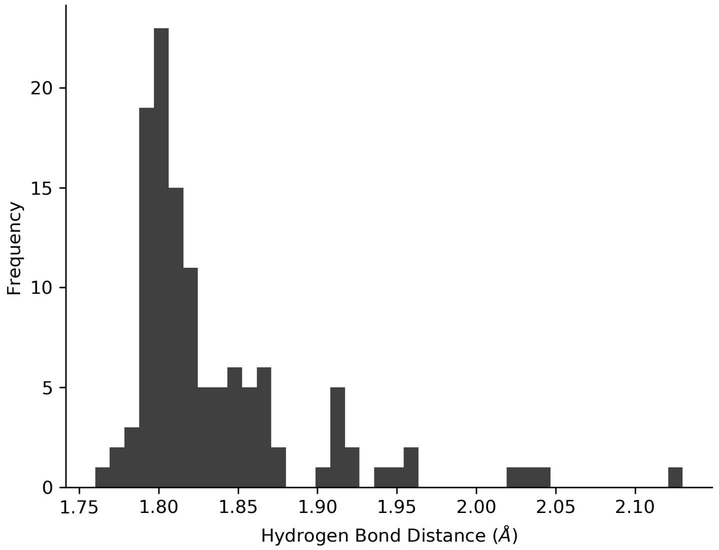

Figure 26: Hydrogen bond distance distribution (Å) for imidazoles (119 data points).

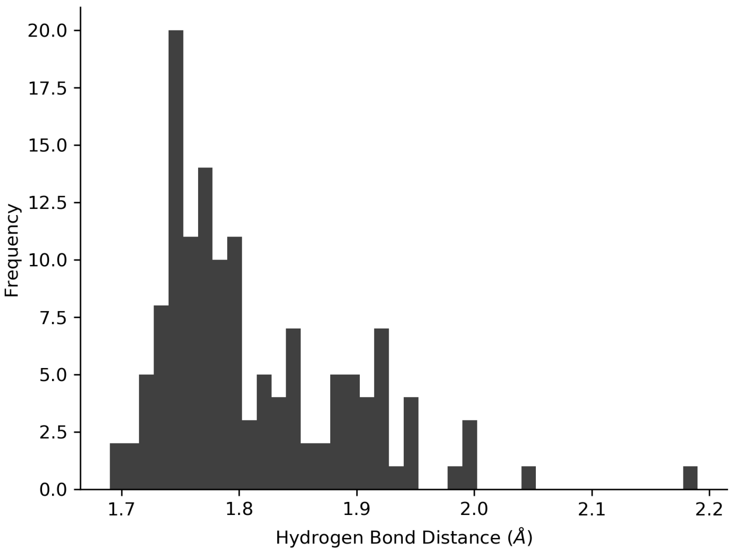

Figure 27: Hydrogen bond distance distribution (Å) for imines (138 data points).

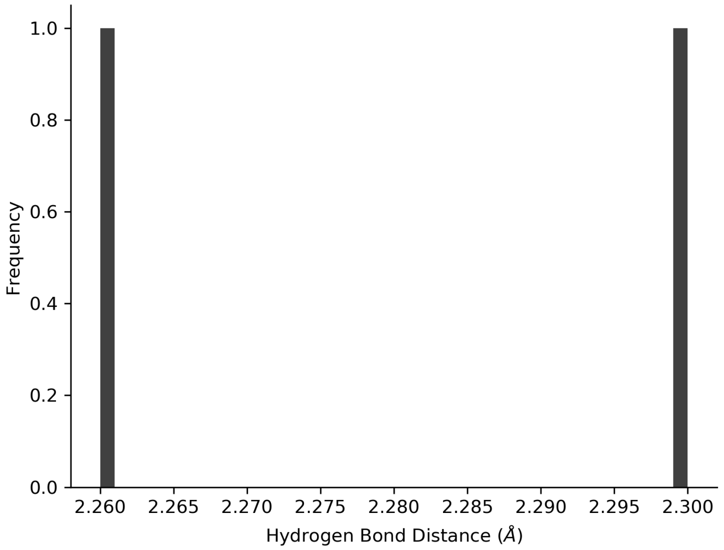

Figure 28: Hydrogen bond distance distribution (Å) for nitrates (2 data points).

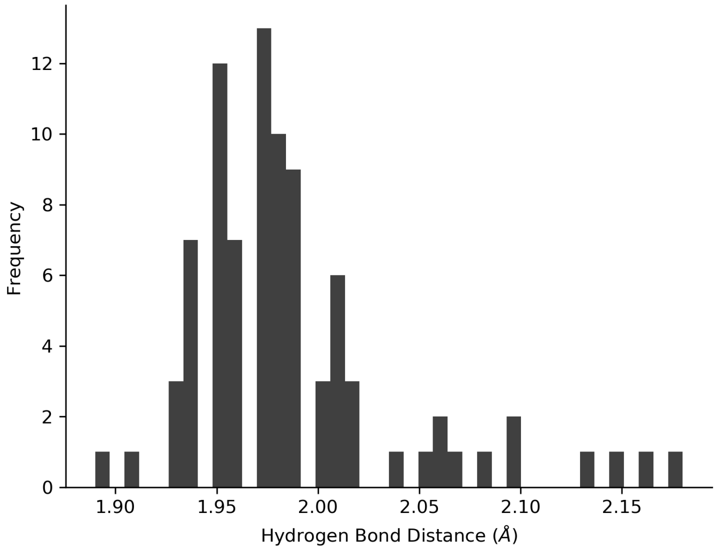

Figure 29: Hydrogen bond distance distribution (Å) for nitriles (87 data points).

ether-HBdistance-distribution.png

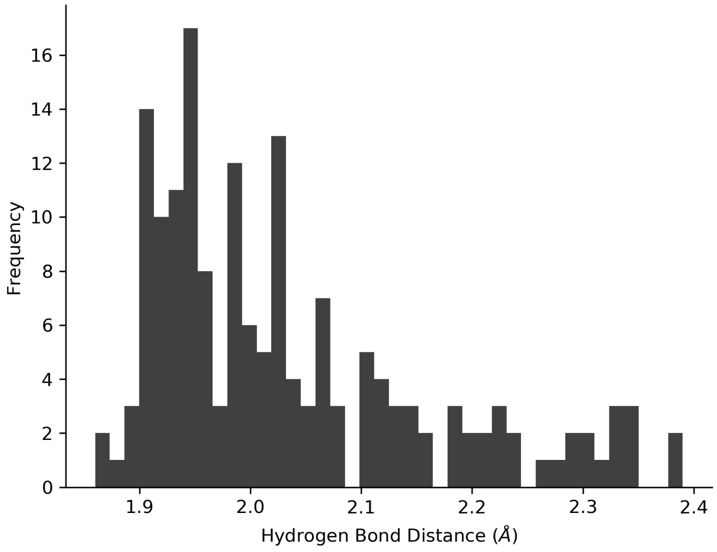

Figure 30: Hydrogen bond distance distribution (Å) for phenol ethers (166 data points).

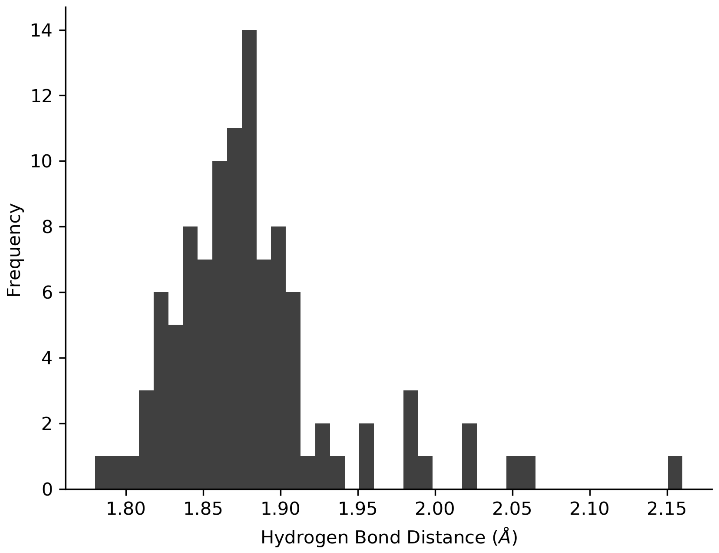

Figure 31: Hydrogen bond distance distribution (Å) for pyrazoles (103 data points).

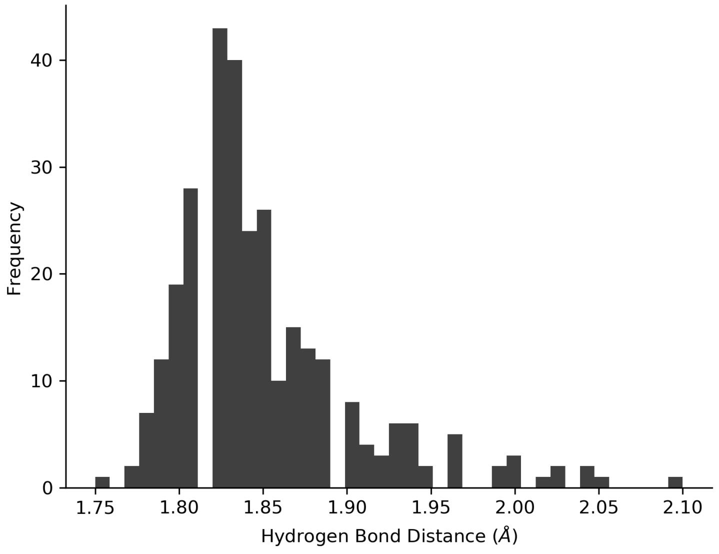

Figure 32: Hydrogen bond distance distribution (Å) for pyridines (298 data points).

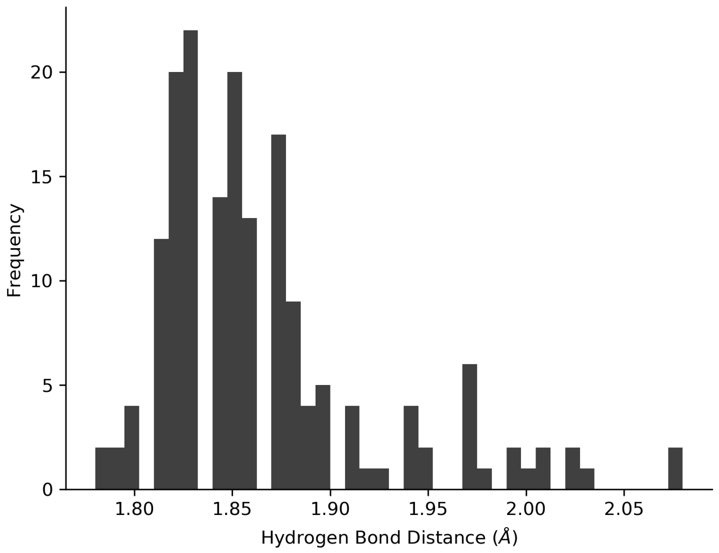

Figure 33: Hydrogen bond distance distribution (Å) for pyrimidines (173 data points).

amine-HBdistance-distribution.png

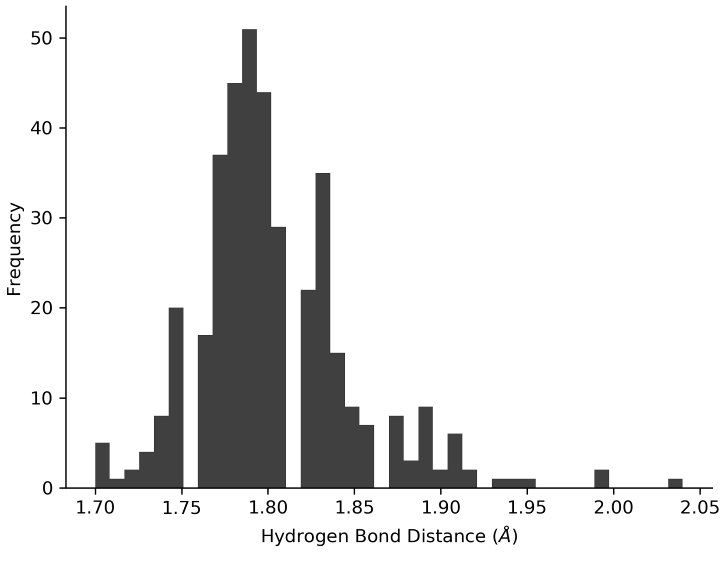

Figure 34: Hydrogen bond distance distribution (Å) for secondary amines (387 data points).

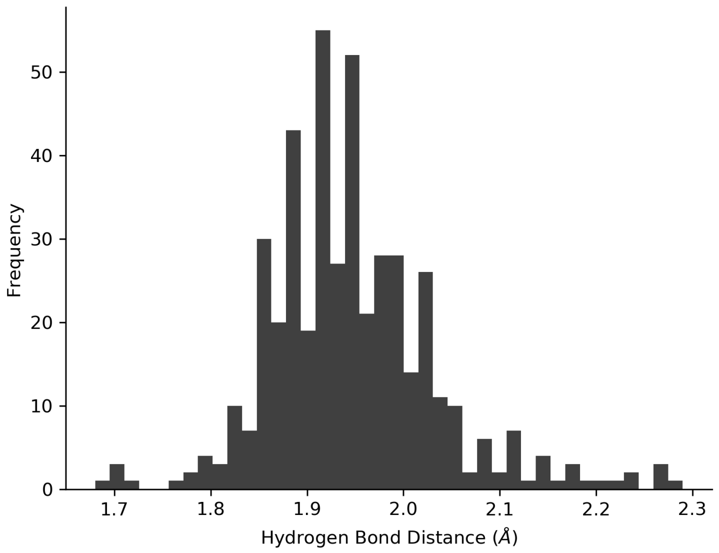

Figure 35: Hydrogen bond distance distribution (Å) for sulfinyls (451 data points).

amine-HBdistance-distribution.png

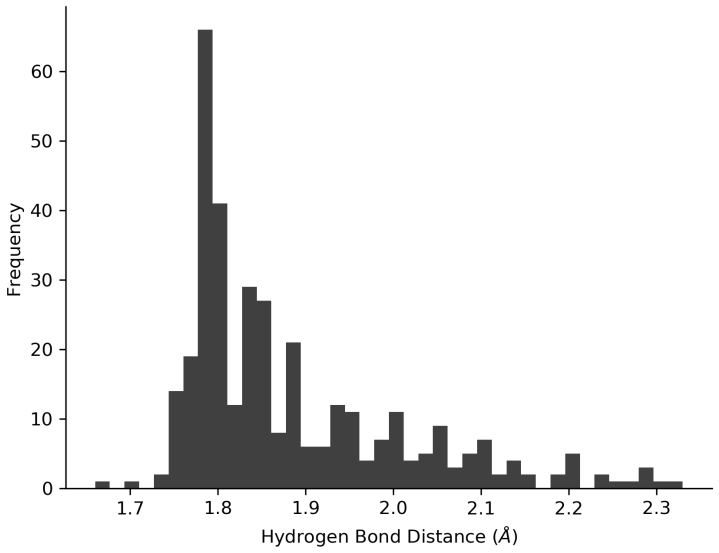

Figure 36: Hydrogen bond distance distribution (Å) for tertiary amines (355 data points).

acceptor function-HBdistance-distribution.png

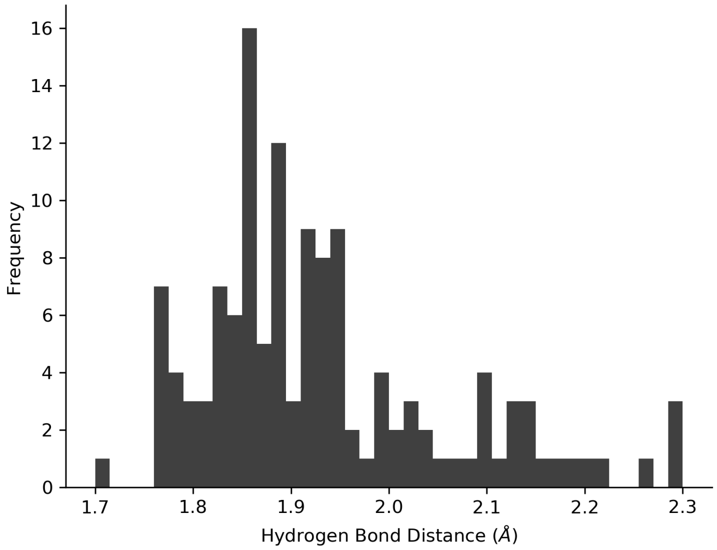

Figure 37: Hydrogen bond distance distribution (Å) for undefined acceptor functions (130 data points).
