# Supplementary material for: Machine learning models for hydrogen bond donor and acceptor strengths using large and diverse training data generated by first-principles interaction free energies
Source: J Cheminform. 2019 Sep 11;11:59. doi: 10.1186/s13321-019-0381-4 (PMC6737620; doi:10.1186/s13321-019-0381-4)
Supplement: Supplementary file 7 — Additional file 7. Documentation for the HBD database. [file 13321_2019_381_MOESM7_ESM.pdf]

The QC Hydrogen Bond Donors Database Documentation

Contents

1 Structures and Methods **3**
1.1 Fragment Generation and Selection 3
1.2 Quantum Chemical Method 4

2 Properties **5**
2.1 E_el 5
2.2 HBD_atom_index 5
2.3 Function 5
2.4 Atom 6
2.5 Mol_ID 6
2.6 E_el_PW6B95 6
2.7 E_el_PBEh-3c 6
2.8 E_el complex_PW6B95 6
2.9 E_el complex_PBEh-3c 6
2.10 HB_distance 6
2.11 dG(298)_RRHO (kJ/mol) 7
2.12 dG(298)_RRHO complex (kJ/mol) 7
2.13 dG_solv_SMD_CCl4 (kJ/mol) 7
2.14 dG_solv_SMD_CCl4 complex (kJ/mol) 7
2.15 Delta G_sol (kJ/mol) 7
2.16 Delta G (kJ/mol) 7
2.17 3D Complex Structures 8

3 Statistics **9**
3.1 Distributions of Target Values (kJ mol^{-1}) 9
3.2 Distributions of HB Distances 17
3.3 Correlation of HB Distances with Target Values 24

1 Structures and Methods

The QC_donors database holds quantum chemically computed hydrogen bond donor strengths, assigned to hydrogen bond donor (HBD) atoms. 737 2D molecular structures are stored in the database. They originate from 276,004 ChEMBL23 active compounds and represent hydrogen bond donor moieties. Each structure may have a single or multiple donor sites. The donor atoms are carbons (in case of alkynes), nitrogens, oxygens and thiolic sulfurs.

1.1 Fragment Generation and Selection

The strategy to generate the fragment structures is as follows:

1. Define hydrogen bond donor sites: R-OH, R-SH, R_2-NH, R-NH_2, R-C≡C-H.
2. Iterate over all donor sites: Get the atom environment (substructure) up to the 4th shell. Three cases are defined then:
   (a) Chain fragment: Atoms around the HBD site are not in any ring up to the third shell. If fourth shell atoms are in a ring, the atom type is changed to an sp^3 atom.
   (b) Ring + side chain fragment: At least one oatom within the third shell around the HBD site is part of a ring. The whole ring is taken in addition to the side chain, which extends to the fourth shell.
   (c) Ring fragment: The HBD site is in a ring. The whole rings system and any side chains up to the fourth shell are taken.

Figure 1: Fragmentation strategy to arrive at the molecular structures

Unique fragments are thus generated. Structures are discarded (filtered) by the following criteria:

- all inorganic structures
- all phosphorous containing structures
- all structures with a corrected molecular weight (MW_{corr} = MW with all halogen atoms given the mass of flourine atoms) $>$ 300.
- all structures with more than three rings

1,088 structures were then selected by the following method:

The clustering/distance to clusters method needs to be described here! . Quantum chemical calculations were performed on these structures and on generated complexes with the reference hydrogen bond acceptor acetone. 737 structures had at least one complete set of successful quantum chemical computations, and thus at least one computed pK_{BHX} value.

1.2 Quantum Chemical Method

The computation of hydrogen bond acceptor strengths by quantum chemistry consisted of the following protocol:

Protocol for generating 3D structures of the acceptor molecules and 3D structures of the complexes with acetone as the hydrogen bond donor:

1. Add hydrogens to the 2D structure by the rdkit, Version 2017.09.1. The tautomer structure is determined by the bond block. The bond orders were taken in the kekulized form as provided by the original ChEMBL23 actives data set.

2. Generate one 3D structure (conformer) by the ETKDG method as implemented by Riniker and Landrum in the rdkit, Version 2017.09.1.

3. Find hydrogen bond acceptor sites.

4. Move the donated hydrogen to the origin and place the hydrogen bond donor atom along the z axis. Rotate the rest of the molecule accordingly.

5. Place the lone pair charge center of the reference acceptor acetone at 2 Ådistance from the donated hydrogen at an angle of 180°.

6. Optimize the structure for 100 steps using the MMFF94s force field as implemented in the rdkit, Version 2017.09.1 by Landrum. The hydrogen bond distance is constrained to 2 Å. The hydrogen bond angle is constrained to 180°.

Protocol for computing the hydrogen bond donor strength quantum chemically:

1. Optimize the 3D structures of the donor molecules and the complexes at the PBEh-3c level of theory using TURBOMOLE, version 7.0.2.

2. Compute the harmonic frequencies of the donor molecules and the complexes at the PBEh-3c level of theory using TURBOMOLE, version 7.0.2.

3. Compute the single point energies of the donor molecules and the complexes at the PW6B95-D3(BJ)/def2-QZVP level of theory using TURBOMOLE, version 7.0.2.

4. Compute the solvation free energies of the donor molecules and the complexes at the SMD(BP86/def2-TZVP) level of theory for the solvent CCl_4 using Gaussian 09.

All the quantum chemically determined energies for the donor molecules and their complexes are reported in the database. The energies for the hydrogen bond acceptor molecule acetone are as follows:

Table 1: Energies of acetone optimized at the PBEh-3c level of theory.

	energy
E (PW6B95-D3(BJ)/def2-QZVP)	-193.479707147 E_h
G (RRHO correction)	153.11 kJ mol^{-1}
δG_solv (CCl_4)	-17.75 kJ mol^{-1}

2 Properties

All properties contained by the molecules in the SDF file are described in this section.

2.1 E_el

This is the electronic energy at the PBEh-3c level of theory

2.2 HBD_atom_index

These are the indices of the HBD. There is a new line character after every index if there is more than one acceptor site in the structure.

2.3 Function

These are the functional groups as determined by rdkit substructre matching. Statistics about the functional groups are found in section 3. There is a new line character after every functional group if there is more than one acceptor site in the structure. If the donor function could not be determined by the algorithm, 'undefined donor function' is placed.

2.4 Atom

This can be 'N', 'O', 'C', and 'S'. If there is more than one donor site in the structure, they are separated by new line characters.

2.5 Mol_ID

This is the identifier. It indicates which ChEMBL molecule the fragment originates from.

2.6 E_el_PW6B95

This is the electronic energy at the PW6B95-D3(BJ)/def2-QZVP level of theory for the structure optimized at the PBEh-3c level of theory. This value is reported in E_{h}.

2.7 E_el_PBEh-3c

This is the electronic energy at the PBEh-3c level of theory for the structure optimized at the PBEh-3c level of theory. This value is reported in E_{h}.

2.8 E_el complex_PW6B95

This is the electronic energy at the PW6B95-D3(BJ)/def2-QZVP level of theory for the complex with acetone optimized at the PBEh-3c level of theory. This value is reported in E_{h}. If there is more than one complex, one value is reported for each complex, separated by new line characters.

2.9 E_el complex_PBEh-3c

This is the electronic energy at the PW6B95-D3(BJ)/def2-QZVP level of theory for the complex with acetone optimized at the PBEh-3c level of theory. This value is reported in E_{h}. If there is more than one complex, one value is reported for each complex, separated by new line characters.

2.10 HB_distance

This is the hydrogen bond distance of the 3D complex structure with acetone as the hydrogen bond donor in Å, optimized at the PBEh-3c level of theory.

2.11 dG(298)_RRHO (kJ/mol)

This is the thermal correction to the free energy in the gas phase as computed by the rigid rotor harmonic oscillator approximation for the structure optimized at the PBEh-3c level of theory.

2.12 dG(298)_RRHO complex (kJ/mol)

This is the thermal correction to the free energy in the gas phase as computed by the rigid rotor harmonic oscillator approximation for the complex with acetone optimized at the PBEh-3c level of theory. If there is more than one complex, one value is reported for each complex, separated by new line characters.

2.13 dG_solv_SMD_CCl4 (kJ/mol)

This is the solvation free energy as computed by the SMD(BP86/def2-TZVP) level of theory for the structure optimized at the PBEh-3c level of theory.

2.14 dG_solv_SMD_CCl4 complex (kJ/mol)

This is the solvation free energy as computed by the SMD(BP86/def2-TZVP) level of theory for the complex with acetone optimized at the PBEh-3c level of theory. If there is more than one complex, one value is reported for each complex, separated by new line characters.

2.15 Delta G_sol (kJ/mol)

This is the computed ΔG_{sol} value as given by the formula:

$$\Delta G_{\text{sol}} = G_{\text{sol}}(\text{complex}) - G_{\text{sol}}(\text{acetone}) - G_{\text{sol}}(\text{acceptormolecule}) \tag{1}$$

If there is more than one complex, one value is reported for each complex, separated by new line characters.

2.16 Delta G (kJ/mol)

The target value in kJ/mol. It is computed by the following linear model from Delta G_sol (kJ/mol):

$$\Delta G = 0.63 \times \Delta G_{\text{sol,QC}} - 20.94 \text{ kJ mol}^{-1} \tag{2}$$

2.17 3D Complex Structures

A 3D sdf database is supplied with each 3D complex structure (PBEh-3c optimized coordinates) supplied. All properties above are copied to the 3D sdf accordingly, i.e., the first complex gets the first target value etc. Only the entries with successful QC computations are found in the 3D sdf.

3 Statistics

This section summarizes the data according to the functional groups involved. The means (μ) for the hydrogen bond distances (Å) and target values (HBD strengths, kJ mol^{-1}) are shown in Table 2.

Table 2: QC donors database analysis by functional groups. .

function	atom	data points	μ(HB distance)/Å	$\mu(\Delta G)$/kJ mol^{-1}
alcohol	O	275	1.91	-4.17
alkyne	C	6	2.38	3.37
amide	N	300	2.13	-2.61
aniline	N	107	2.19	-2.58
imine	N	4	2.41	3.15
pyrazole	N	19	2.18	-1.88
pyrrole	N	44	2.04	-3.26
secondary amine	N	121	2.35	2.40
thioamide	N	2	2.11	0.68
thiol	S	17	2.47	3.71
undefined donor	N/O	141	2.12	-1.56
total	C/N/O/S	1036	2.11	-2.08

3.1 Distributions of Target Values (kJ mol^{-1})

The distribution of all HBD strengths (ΔG for acetone complex formation) is shown in Fig. 14

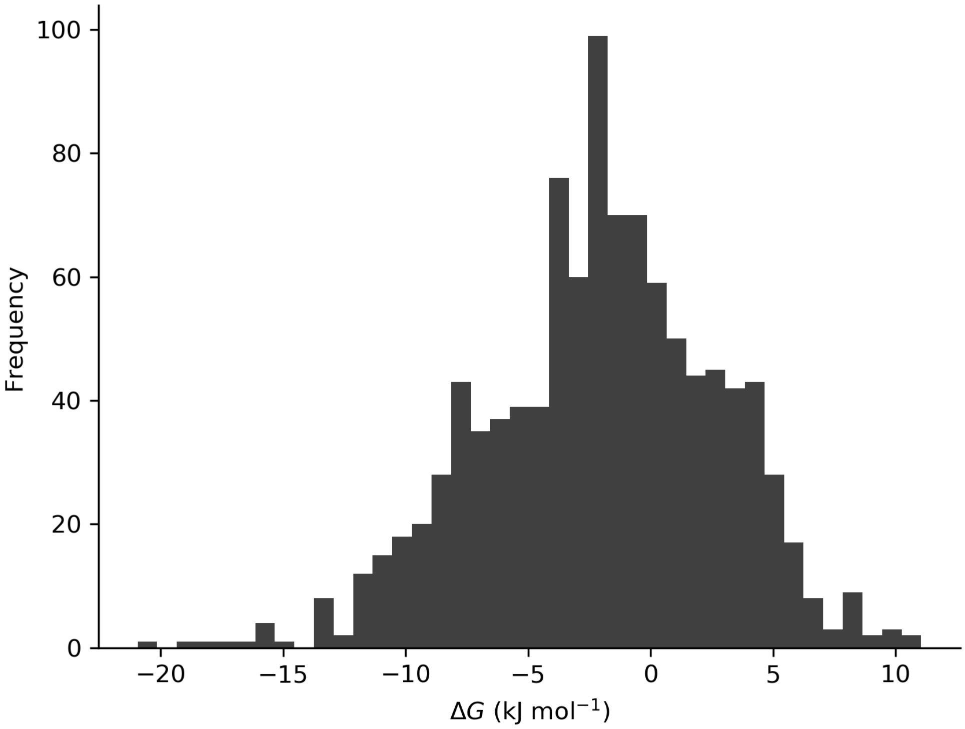

Figure 2: HBD strengths (ΔG for acetone complex formation) for the total QC_donors database (1036 data points).

The following figures contain the distributions of target values by functional groups within the database.

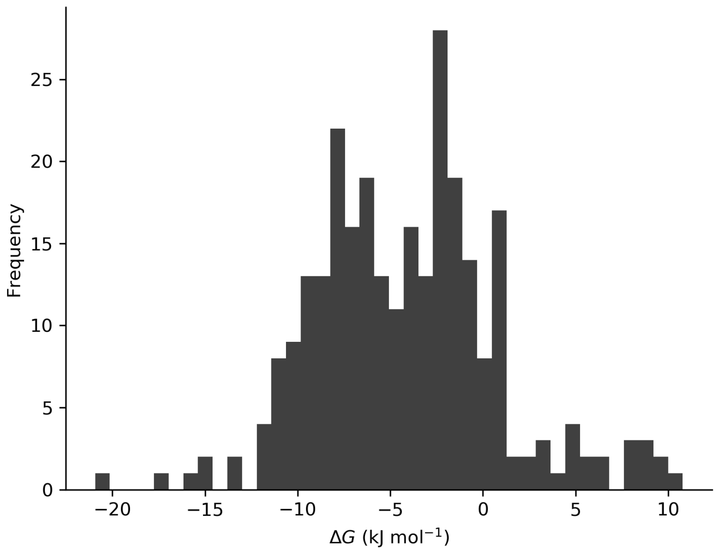

Figure 3: HBD strengths (ΔG for acetone complex formation) for alcohols (275 data points).

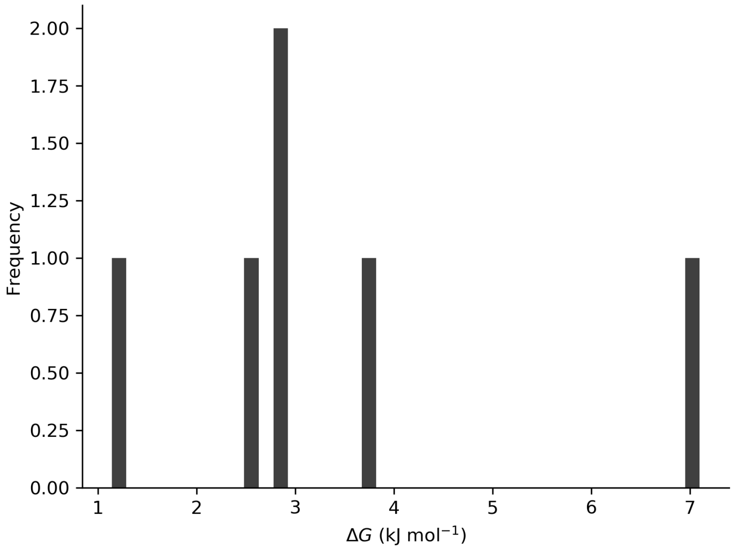

Figure 4: HBD strengths (ΔG for acetone complex formation) for alkynes (6 data points).

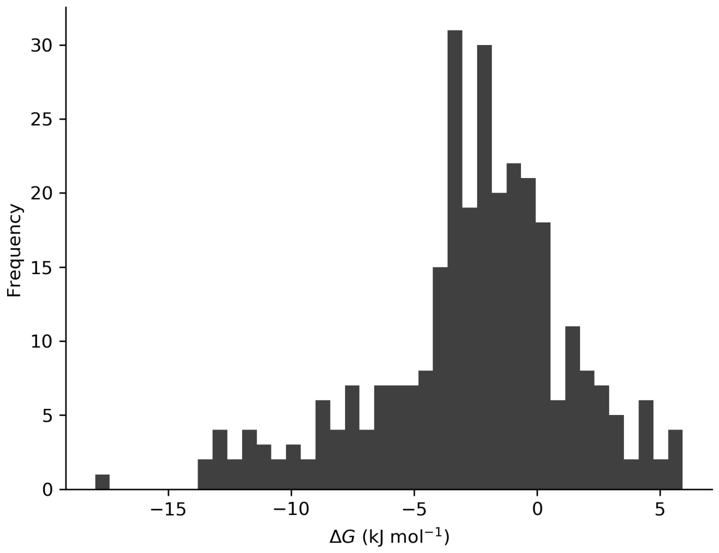

Figure 5: HBD strengths (ΔG for acetone complex formation) for amides (300 data points).

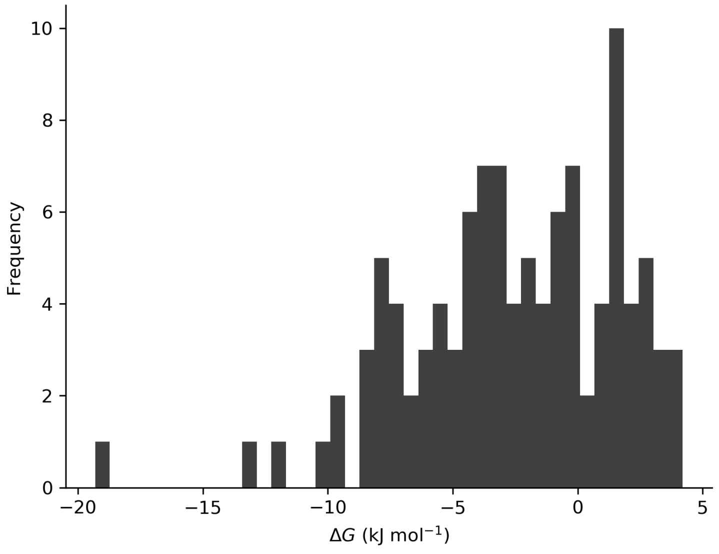

Figure 6: HBD strengths (ΔG for acetone complex formation) for anilines (107 data points).

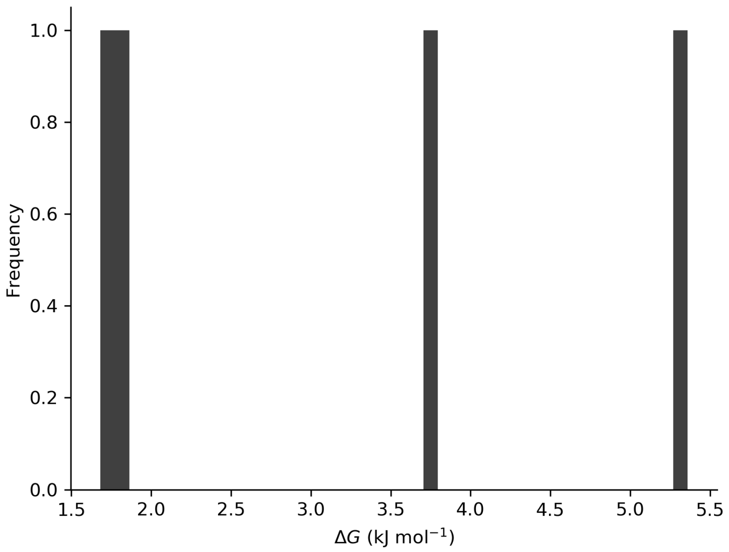

Figure 7: HBD strengths (ΔG for acetone complex formation) for imines (4 data points).

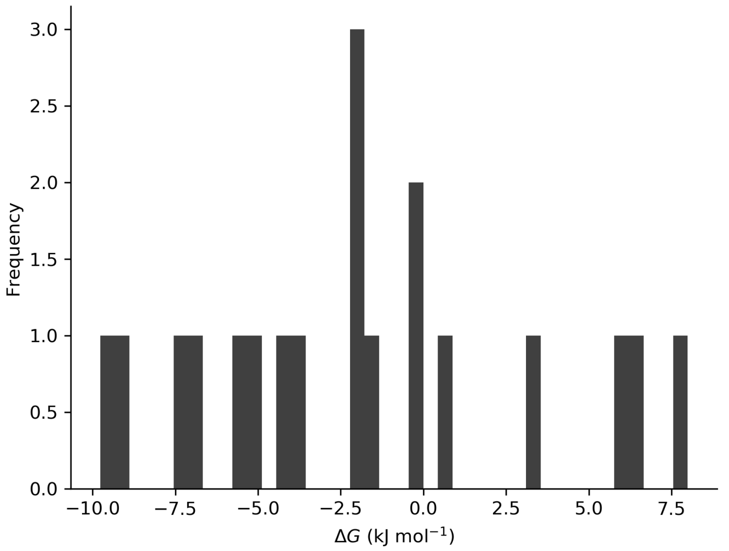

Figure 8: HBD strengths (ΔG for acetone complex formation) for pyrazoles (19 data points).

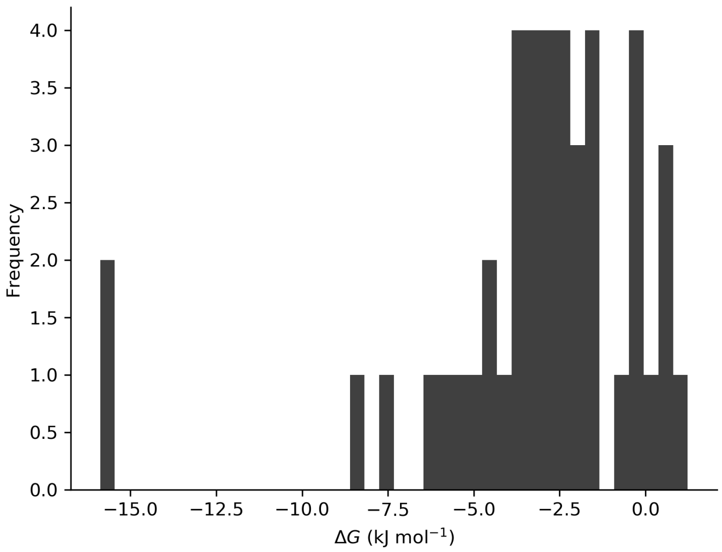

Figure 9: HBD strengths (ΔG for acetone complex formation) for pyrroles (44 data points).

amine-targetvalue-distribution-kJoules.png

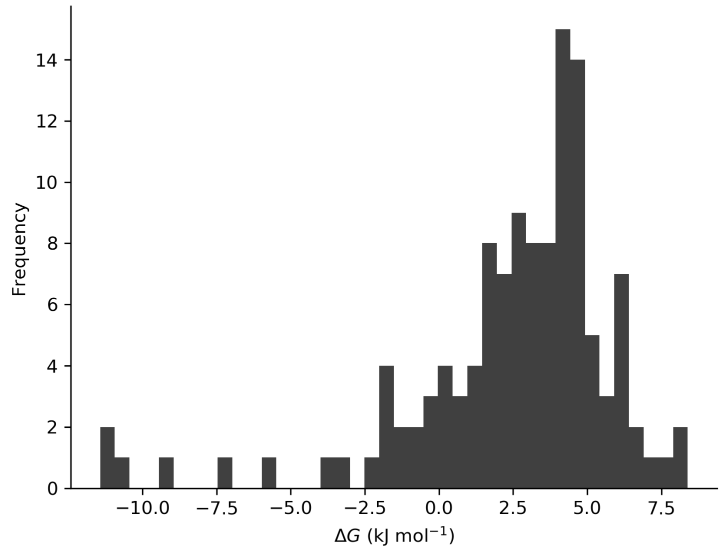

Figure 10: HBD strengths (ΔG for acetone complex formation) for secondary amines (121 data points).

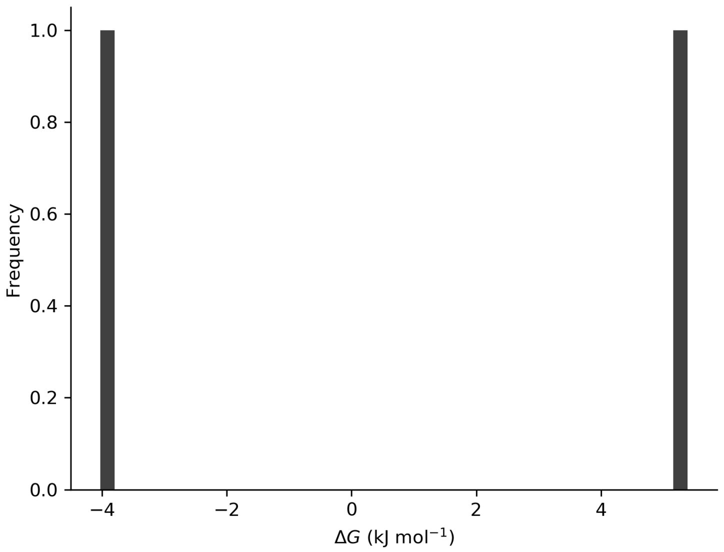

Figure 11: HBD strengths (ΔG for acetone complex formation) for thioamides (2 data points).

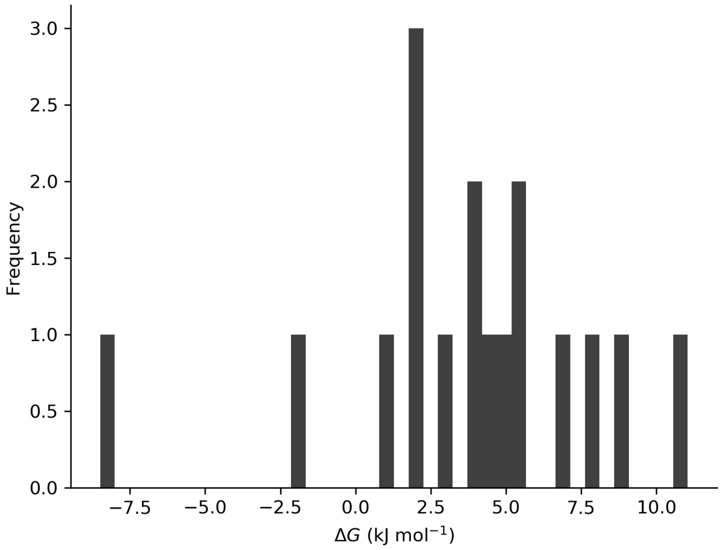

Figure 12: HBD strengths (ΔG for acetone complex formation) for thiols (17 data points).

donor function-targetvalue-distribution-kJoules.png

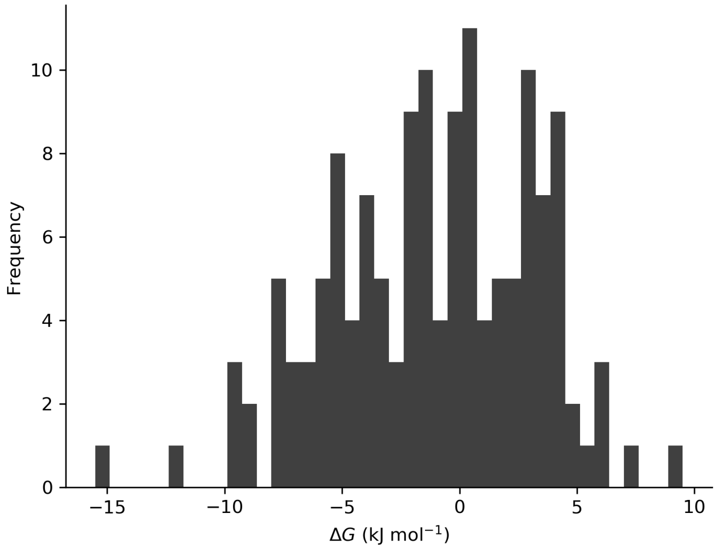

Figure 13: HBD strengths (ΔG for acetone complex formation) for undefined donor functions (141 data points).

3.2 Distributions of HB Distances

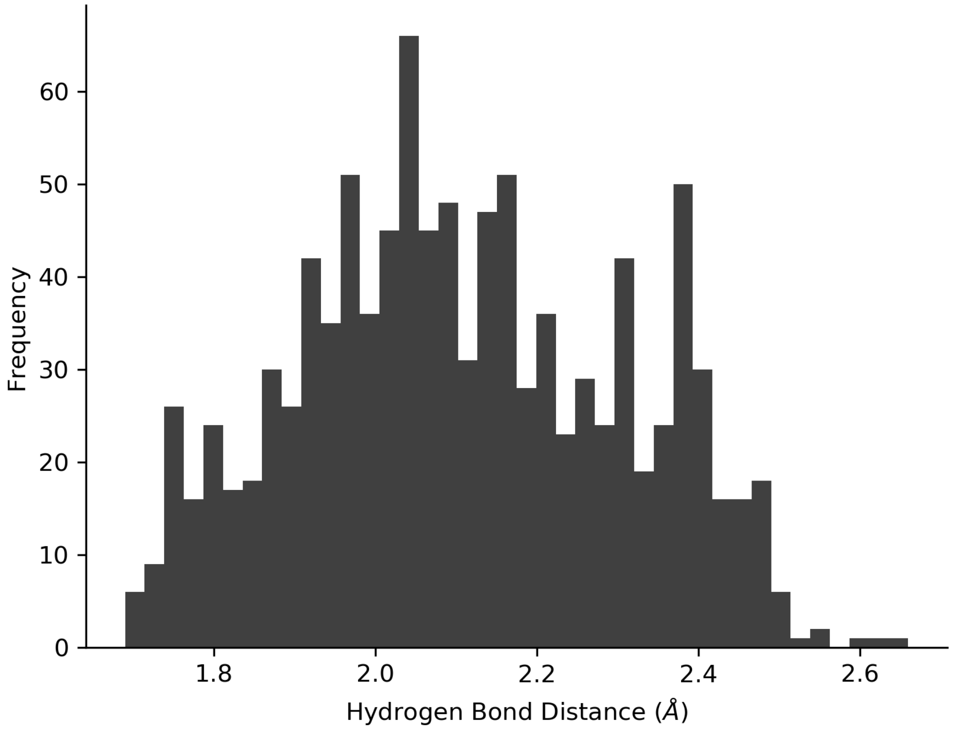

Figure 14: Hydrogen bond distance distribution (Å) for the total QC_donors database (4426 data points).

The following figures contain the distributions of hydrogen bond distances by functional groups within the database.

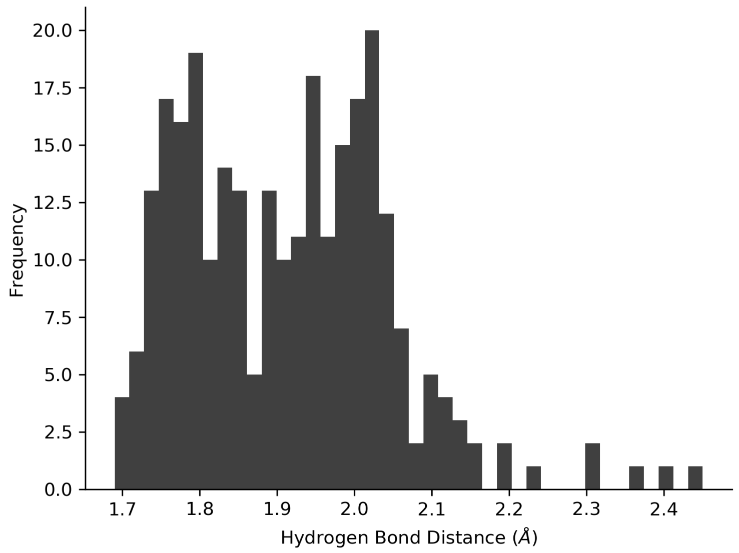

Figure 15: Hydrogen bond distance distribution (Å) for alcohols (275 data points).

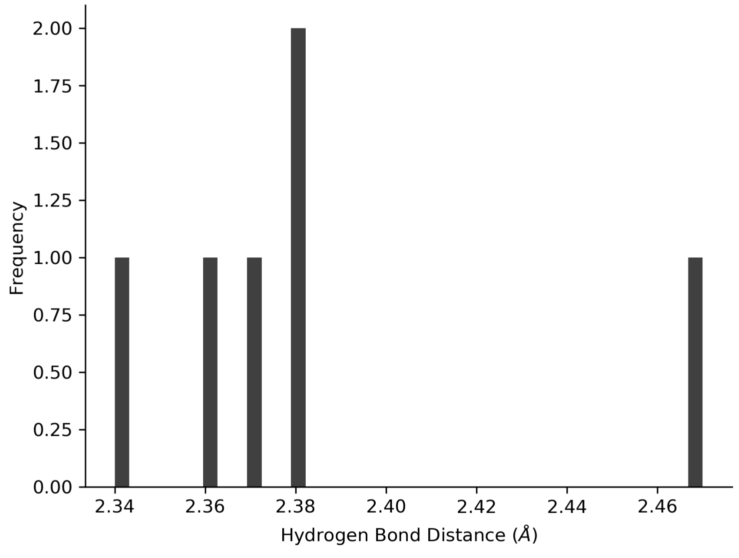

Figure 16: Hydrogen bond distance distribution (Å) for alkynes (6 data points).

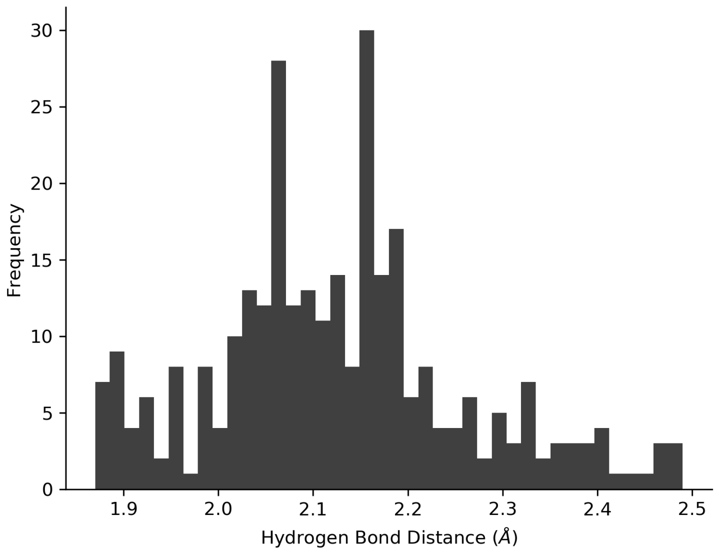

Figure 17: Hydrogen bond distance distribution (Å) for amides (300 data points).

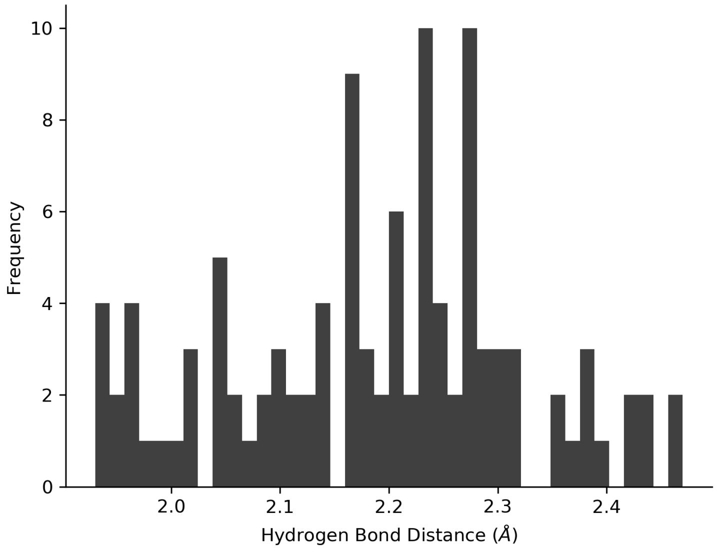

Figure 18: Hydrogen bond distance distribution (Å) for aniline (107 data points).

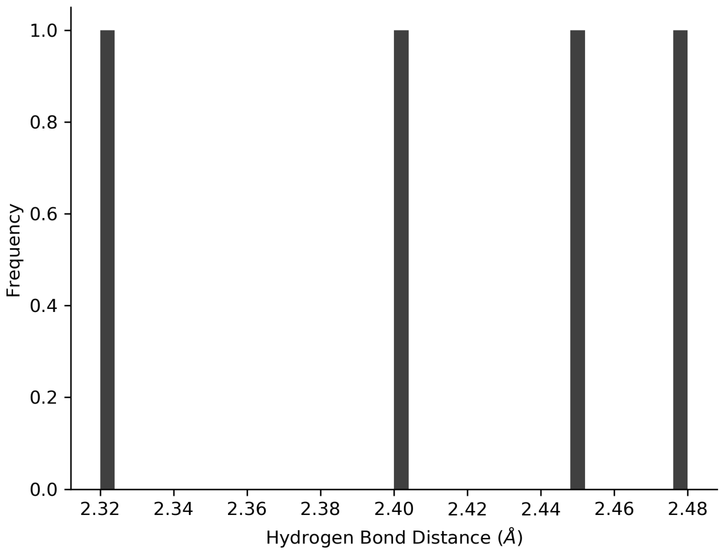

Figure 19: Hydrogen bond distance distribution (Å) for imines (4 data points).

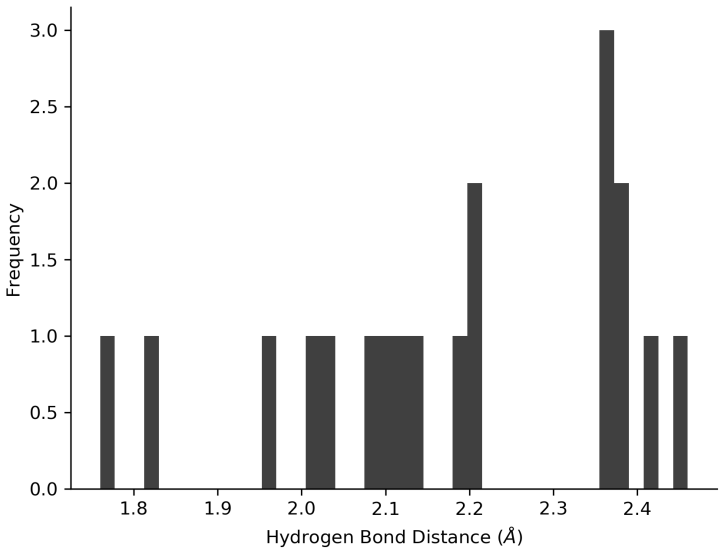

Figure 20: Hydrogen bond distance distribution (Å) for pyrazoles (19 data points).

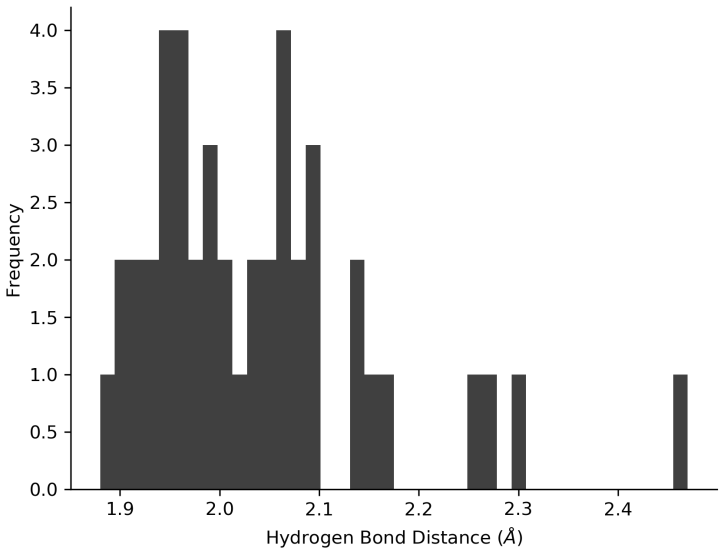

Figure 21: Hydrogen bond distance distribution (Å) for pyrroles (44 data points).

amine-HBdistance-distribution.png

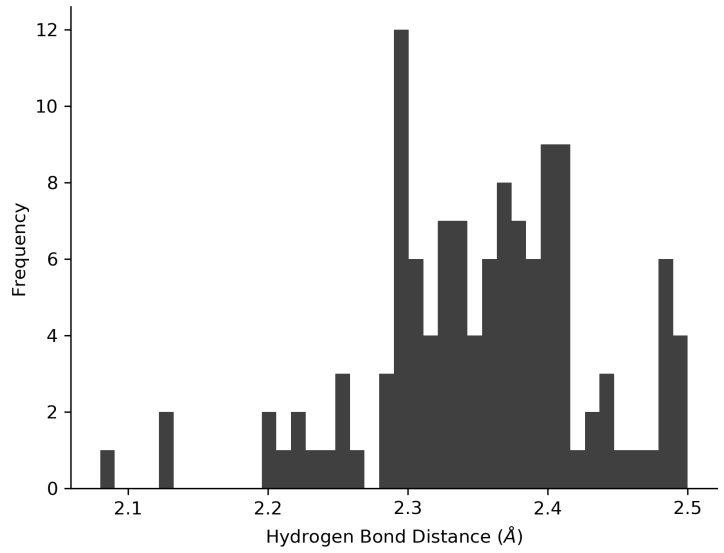

Figure 22: Hydrogen bond distance distribution (Å) for secondary amines (121 data points).

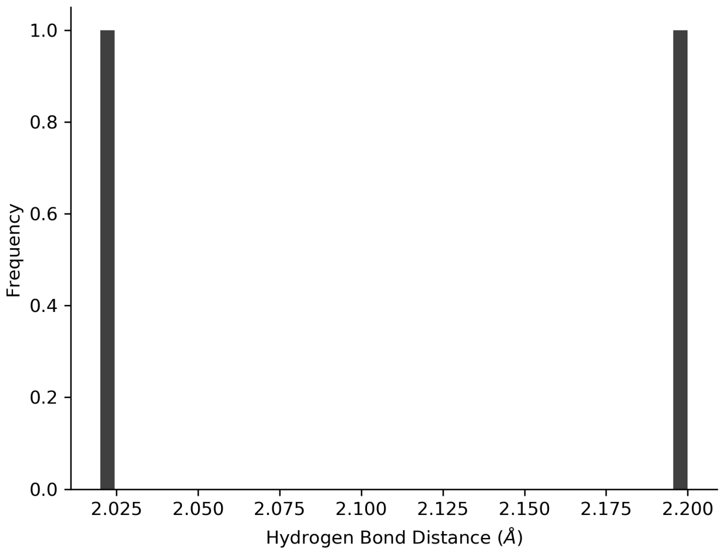

Figure 23: Hydrogen bond distance distribution (Å) for thioamides (2 data points).

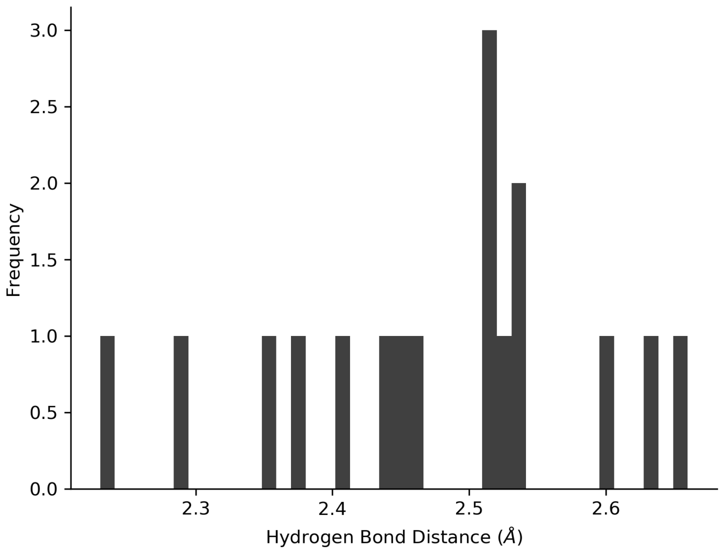

Figure 24: Hydrogen bond distance distribution (Å) for thiols (17 data points).

donor function-HBdistance-distribution.png

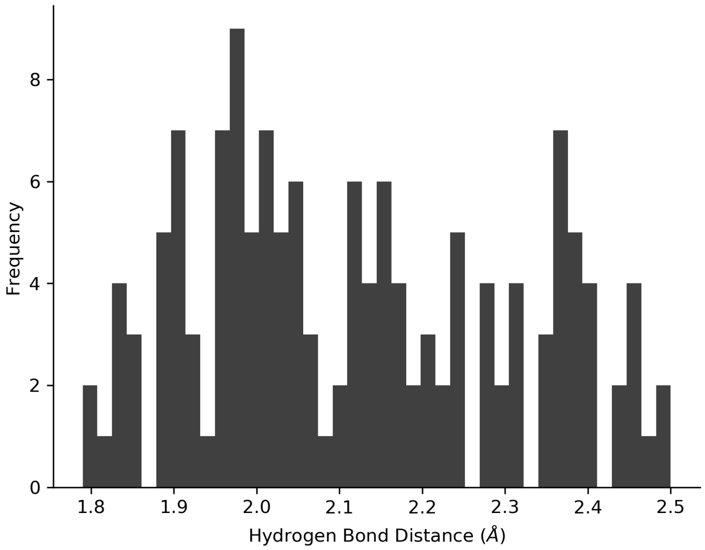

Figure 25: Hydrogen bond distance distribution (Å) for undefined donor functions (141 data points).
